# Systematic Optimization of Sputtered AZO Thin Films and Their Integration as Alternative Transparent Electrodes for Perovskite Solar Cells

**DOI:** 10.1021/acsomega.6c06694

**Published:** 2026-08-21

**Authors:** Zeynep Mavili, Mucahit Yilmaz

**Affiliations:** † Laboratory of Advanced Materials & Photovoltaics (LAMPs), Necmettin Erbakan University, Konya 42090, Türkiye; § Department of Basic Sciences, Necmettin Erbakan University, Konya 42090, Türkiye; ∥ Department of Nanoscience and Nanoengineering, Institute of Science, Necmettin Erbakan University, Konya 42090, Türkiye

## Abstract

Transparent conducting oxides (TCOs) are critical components of perovskite solar cells (PSCs), motivating the search for low-cost and scalable alternatives to conventional fluorine-doped tin oxide (FTO). Among the proposed alternatives, Al-doped ZnO (AZO) has been widely investigated as a low-cost, indium-free transparent conductive oxide. However, systematic studies correlating sputtering parameters with AZO thin film properties and PSC performance through direct benchmarking against commercial FTO under identical device fabrication conditions remain limited. In this study, AZO thin films were systematically optimized by magnetron sputtering through controlled variation of sputtering power, annealing conditions, substrate temperature, and deposition duration. The optimized high-temperature-grown AZO film (AZO-13) exhibited high visible transparency (∼86%), low sheet resistance (1.26 × 10^1^ Ω·sq^–1^), strong preferential (002) orientation, and high calculated electrical conductivity (∼9.85 × 10^2^ S·cm^–1^), achieving a Haacke figure of merit (0.668) that slightly surpassed that of commercial FTO (0.664). To evaluate its applicability as a transparent electrode, the optimized AZO film was directly integrated into PSC devices and compared with commercial FTO under identical fabrication conditions. The AZO-based PSC achieved a stabilized efficiency of 16.49%, while comparative PL/TRPL, EQE, and EIS analyses revealed that the lower device performance mainly originated from increased interfacial resistance and less efficient charge extraction. In addition, AZO exhibited improved long-wavelength transparency compared with FTO, suggesting potential relevance for tandem-oriented photovoltaic applications. These results establish a clear process–structure–property–device relationship for sputtered AZO thin films and demonstrate their potential as alternative TCO electrodes for PSC technologies.

## Graphical abstract

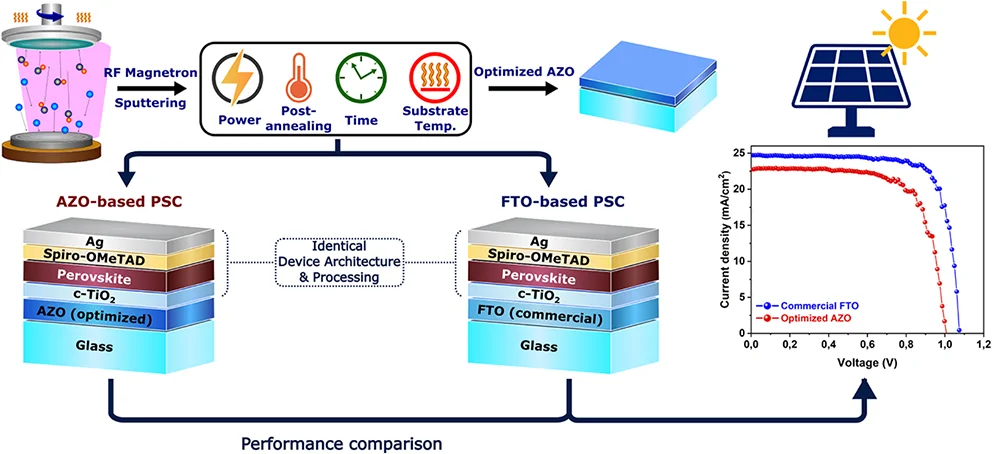

## Introduction

Transparent conducting oxides (TCOs) constitute a unique class of materials that simultaneously exhibit high optical transparency in the visible spectrum and high electrical conductivity, making them indispensable in modern optoelectronic technologies.^1^ This remarkable combination of properties enables TCOs to function as transparent electrodes in a wide range of applications where efficient light transmission and charge transport are simultaneously required. These applications include photovoltaic devices, flat-panel displays, touchscreen technologies, optoelectronic devices, smart windows, transparent heaters, and sensor systems.^2–14^ Among the TCO family, fluorine-doped tin oxide (FTO) and indium tin oxide (ITO) remain the most widely used materials due to their well-established processing techniques and reliable performance. However, the growing demand for cost reduction, material sustainability, and compatibility with low-temperature processing has driven the search for alternative TCO materials.^15,16^ In this context, aluminum-doped zinc oxide (AZO) has emerged as one of the most promising candidates due to its earth abundance, nontoxicity, and competitive optoelectronic properties. ZnO thin films doped with aluminum (typically 1–5%) can achieve carrier concentrations on the order of 10^20^ cm^–3^, resulting in low resistivity and relatively high carrier mobility.^17^ However, excessive doping may introduce additional scattering centers, leading to reduced mobility and degraded conductivity. The intrinsic bandgap of AZO lies in the range of approximately 3.2–3.4 eV, and depending on the doping level, a blue shift associated with the Moss–Burstein effect can extend the apparent bandgap up to ∼4.1 eV.^18^ These characteristics enhance its suitability as a transparent electrode in optoelectronic devices. Recent studies have reported that AZO thin films can achieve optical transmittance exceeding 85% in the visible region and resistivity values on the order of 10^–4^ Ω·cm under optimized deposition conditions.^19,20^ Furthermore, recent advances in defect engineering and dopant activation have demonstrated additional improvements in carrier mobility and reduced scattering losses.^21,22^

Photovoltaic technologies currently represent one of the major research directions for sustainable energy production, and among emerging photovoltaic systems, perovskite solar cells (PSCs) have attracted considerable attention because of their rapidly increasing power conversion efficiencies and low-cost fabrication potential. These advances have stimulated increasing interest in developing transparent conductive electrodes that combine high optical transparency, low electrical resistivity, long-term stability, and compatibility with scalable manufacturing technologies.^23^ In PSC architectures, TCOs play a critical role as electrodes by simultaneously enabling efficient light transmission and charge extraction, and, besides functioning as transparent current collectors, they critically influence optical coupling, interfacial recombination, and ultimately the efficiency and operational stability of PSC devices. Therefore, the development of alternative TCO materials with improved optoelectronic properties and scalable fabrication compatibility has become increasingly important for next-generation PSC technologies,^24^ and significant research efforts have consequently been devoted to developing indium-free TCO materials and optimizing their composition, microstructure, and interface properties for next-generation photovoltaic applications.^25,26^ These features make AZO a relevant candidate for PSC-oriented transparent electrode development. Recent studies have indeed identified AZO as one of the most promising indium-free TCO candidates because of its earth-abundant constituent elements, low material cost, and compatibility with scalable, large-area deposition techniques. Nevertheless, further optimization of film properties and more systematic validation at the photovoltaic device level are still required before its full technological potential can be realized.^27–29^

Despite the promising optoelectronic properties of AZO, several important limitations remain in the literature. Most studies primarily focus on material-level optimization and report improvements in transparency, resistivity, and crystallinity without systematically evaluating how these changes influence device-level photovoltaic performance. Since the structural and electrical properties of the transparent conductive electrode directly affect charge transport, carrier extraction, and optical losses in PSCs, understanding the relationship between AZO thin-film properties and key PSC performance parameters, such as power conversion efficiency (PCE), fill factor (FF), and open-circuit voltage (*V*_OC_), is critically important. However, the correlation between AZO thin-film properties and key PSC performance parameters, such as power conversion efficiency (PCE), fill factor (FF), and open-circuit voltage (*V*_OC_), is still not fully understood.^30,31^ In addition, direct benchmarking against commercial TCOs, particularly FTO, under identical device fabrication conditions remains limited. Many previous reports rely on indirect comparisons or literature-based evaluations, making it difficult to assess the practical applicability of AZO electrodes in PSC architectures.^4,30^ Therefore, systematic studies combining AZO optimization, detailed material characterization, and direct PSC device integration are still needed to clarify the realistic potential and current limitations of the AZO-based transparent electrodes.

AZO thin films can be fabricated using a variety of physical and chemical deposition techniques, including magnetron sputtering, mist-chemical vapor deposition (mist-CVD) and CVD, pulsed laser deposition (PLD), atomic layer deposition (ALD), and sol–gel processing.^22,29,32–36^ Among these, magnetron sputtering is particularly advantageous due to its ability to produce dense, uniform, and low-resistivity films with precise control over growth parameters.^32,37^ However, the reported optimum growth conditions for AZO thin films vary considerably among studies, indicating that the underlying process–structure–property relationship has not yet been fully established.^38^ Recent investigations have primarily focused on the use of ALD-grown layers and interconnecting layers in different solar cells and perovskite/Si tandem solar cells,^29,39–42^ whereas comprehensive studies directly correlating optimized AZO growth parameters with PSC device performance remain relatively limited. Systematic investigations correlating sputtering parameters with the resulting structural, optical, and electrical properties of AZO thin films, together with their influence on PSC device behavior, are still relatively limited in the literature.

In this study, AZO thin films were deposited by magnetron sputtering under systematically varied growth conditions. After optimization, the best-performing AZO film was integrated into PSC devices and directly compared to commercial FTO electrodes. The main contribution of this work is the establishment of a direct relationship among sputtering parameters, AZO thin-film properties, and PSC device performance through systematic material optimization and side-by-side benchmarking against commercial FTO under identical device fabrication conditions. This approach provides a comprehensive evaluation of AZO as an alternative TCO for PSC applications.

## Experimental Section

### Optimization of AZO Thin Films

AZO thin films were deposited onto soda-lime glass (SLG) substrates using a VAKSIS MIDAS 3M1T magnetron sputtering system equipped with a commercially available 3-in. ceramic AZO target (Maideli, 98 wt % ZnO and 2 wt % Al_2_O_3_, 99.99% purity). Prior to deposition, the SLG substrates (76 × 26 mm^2^, 1 mm thickness) were cleaned ultrasonically in acetone and isopropyl alcohol, followed by nitrogen drying. Before deposition, the sputtering chamber was evacuated to a base pressure below 5 × 10^–7^ Torr to minimize residual contamination. The AZO films were deposited under systematically varied sputtering conditions to optimize their optoelectronic properties for transparent electrode applications. During all depositions, the working pressure was maintained at approximately 2 × 10^–3^ Torr to minimize pressure-induced variations in plasma conditions. The plasma was ignited at an elevated Ar pressure of approximately 1.0–1.3 × 10^–1^ Torr under a low initial power of 30 W. After ignition, the pressure was gradually reduced to the working pressure of ∼2 × 10^–3^ Torr by progressively throttling the vacuum gate valve. Once the working pressure was stabilized, the sputtering power was subsequently increased in 30 W increments per minute up to the target deposition power. Prior to opening the substrate shutter, the target surface was presputtered for 5 min at the final power to remove surface contaminants and stabilize the plasma. The Ar flow rate was kept constant at 40 sccm, the target-to-substrate distance was fixed at 18 cm, and the substrate holder was continuously rotated at 10 rpm throughout all depositions to ensure consistent plasma exposure and uniform film growth. For the postannealing process, the deposited films were annealed in ambient atmosphere for 60 min. The effects of sputtering power density, postdeposition annealing, substrate temperature, and a combined power–time trade-off at fixed substrate temperature were investigated, as summarized in Table 1. Based on the structural, optical, and electrical characterization results, the optimized AZO electrode was selected for subsequent photovoltaic device fabrication.

**Table 1:** Deposition Parameters Used for the Systematic Optimization of AZO Thin Films^a^

Sample Name	Main Variable	Power (W·cm^–2^)	Substrate Temp. (°C)	Deposition Time (min.)	Post-Annealing Temp. (°C)	Purpose
AZO-1	Sputtering Power	0.5	RT	240	-	Power optimization under approximately comparable thickness conditions
AZO-2		1	RT	120	-	
AZO-3		2	RT	60		
AZO-4		4	RT	30	-	
AZO-5	Postannealing Temp.	4	RT	30	350	Postdeposition annealing optimization
AZO-6		4	RT	30	400	
AZO-7		4	RT	30	450	
AZO-8	Substrate Temp.	6	200	30	-	Thermal growth optimization
AZO-9		6	300	30	-	
AZO-10		6	400	30	-	
AZO-11		6	500	30	-	
AZO-12		6	550	30	-	
AZO-13	Duration/Power	6	500	90	-	Final process optimization
AZO-14		3	500	180	-	

a The optimization process was divided into four stages, including power-dependent growth, post-deposition annealing, substrate-temperature-controlled growth, and final process optimization.

### Perovskite Solar Cell Fabrication

AZO thin films exhibiting the optimum optoelectronic properties were selected as transparent conductive electrodes for PSC fabrication, while commercial FTO-coated SLG substrates (Sigma-Aldrich #735167) were used as reference electrodes. The conductive AZO and FTO layers were patterned using 4 M HCl in the presence of Zn powder to define the device geometry. Prior to deposition, the substrates were ultrasonically cleaned in DI water, Hellmanex solution, acetone, ethanol, and isopropanol, followed by drying under pressurized air flow. Compact TiO_2_ (c-TiO_2_) electron transport layers (ETLs) were deposited onto the substrates by spray pyrolysis using titanium diisopropoxide bis­(acetylacetonate) dissolved in an acetylacetone/ethanol mixture. Before deposition, the substrates were treated by UV–O_3_ for 20 min. The c-TiO_2_ layers were deposited at 450 °C under an oxygen atmosphere and subsequently annealed at 450 °C for 30 min. The perovskite absorber layer with the composition Cs_0.05_(FA_0.85_MA_0.15_)_0.95_Pb­(I_0.85_Br_0.15_)_3_ was deposited using a two-step spin-coating process from a DMF/DMSO (4:1 v/v)-based precursor solution containing CsI, FAI, MABr, PbI_2_, and PbBr_2_, following procedures similar to those reported in our previous studies.^43,44^ During spin coating, 200 μL of chlorobenzene was dynamically dripped onto the rotating substrate to promote rapid crystallization. The films were subsequently annealed at 100 °C for 1 h and cooled under an inert and humidity-controlled atmosphere. Spiro-OMeTAD containing *tert*-butylpyridine (TBP) and lithium bis­(trifluoromethanesulfonyl)­imide (Li-TFSI) additives was used as the hole transport layer (HTL) and spin-coated at 4000 rpm for 30 s. Finally, Ag top electrodes (∼120 nm) were thermally evaporated using a VAKSİS Midas PVD 4O system under 5 × 10^–6^ Torr vacuum through a shadow mask to define the active device area.

### Characterization

The structural properties of the AZO thin films were investigated by grazing-incidence X-ray diffraction (GIXRD, PANalytical EMPYREAN) using Cu Kα radiation (λ = 1.5406 Å) within the 2θ range of 20–80° at a scanning rate of 3 deg·min^–1^. Raman spectroscopy measurements were performed using a Renishaw inVia Confocal Raman system equipped with a 532 nm excitation laser operating at 30 mW. The spectra were collected with an integration time of 1 s and accumulated over 200 scans to improve the signal-to-noise ratio. Surface morphology and microstructural properties were examined using FESEM (ZEISS GeminiSEM 500) and AFM (Park Systems XE7). The surface chemical composition and oxidation states of the AZO thin films were further investigated using a Thermo Scientific K-Alpha scanning XPS microprobe. Optical transmittance spectra were recorded using a SHIMADZU UV-1800 UV–Vis spectrophotometer in the wavelength range of 320–1100 nm, and the optical bandgap values were estimated using Tauc plot analysis. The average visible transmittance was calculated by averaging the measured transmittance values over the wavelength range of 400–800 nm. Film thicknesses were measured using a Profilm 3D optical profilometer. The electrical properties of the AZO thin films, including the sheet resistance (R_S_), were evaluated using a Keithley 2450 SourceMeter in a two-probe configuration, with two parallel Ag electrodes (5 mm separation, 5 mm electrode width, L = W) deposited onto each film and the corresponding current–voltage characteristics recorded under an applied voltage sweep. To verify reproducibility, the optimized deposition condition was repeated in two independent deposition runs, each producing three AZO-coated glass substrates (7.5 × 2.5 cm^2^), and the reported AZO-13 value is representative of the reproducible electrical performance obtained under the optimized deposition conditions. Hall effect measurements were additionally performed at room temperature using a SWin Hall 8800 Hall Effect Measurement System under an applied magnetic field of 7210 G. Electrical contacts were formed using indium at the four corners of 10 mm × 10 mm samples in the standard van der Pauw configuration. To experimentally verify the transport characteristics at different optimization stages, Hall measurements were carried out for representative samples together with the commercial FTO reference. Among these, AZO-1 was deliberately selected as the least-optimized reference point of the series (Stage 1, lowest power density) rather than the best-performing sample of its stage, so that the full extent of the carrier transport improvement achieved through subsequent optimization steps (AZO-7, AZO-11, AZO-13, and AZO-14) could be unambiguously demonstrated. A photograph of the Hall measurement configuration is provided in Figure S1 (Supporting Information), and the measured Hall parameters are summarized in Table S1 (Supporting Information). The photovoltaic performance of the fabricated PSC devices was characterized by current density–voltage (J–V) measurements using a Keithley 4200 SCS parameter analyzer under simulated solar illumination from a 450 W xenon lamp. External quantum efficiency (EQE) measurements were carried out using a PV Measurements QEX10 system. Steady-state and time-resolved photoluminescence (PL/TRPL) measurements were performed using an Edinburgh FS5 spectrofluorometer to investigate charge carrier dynamics and recombination behavior. Electrochemical impedance spectroscopy (EIS) measurements were conducted using an Ivium CompactStat system over the frequency range of 1 MHz–100 Hz to evaluate charge transport and interfacial resistance characteristics of the PSC devices.

## Results and Discussion

### Optimization Strategy of AZO Transparent Electrodes

To identify the most suitable AZO transparent electrode for PSC fabrication, the effects of sputtering power, postdeposition annealing, substrate temperature, and the power–time trade-off were systematically investigated while maintaining the deposition pressure near 2 × 10^–3^ Torr to minimize plasma-related variations. The growth parameters are summarized in Table 1, and the optimization strategy is illustrated in Figure 1. The optimization process consisted of four stages: room-temperature power-dependent growth, postannealing of room-temperature-grown films, substrate-temperature-controlled growth, and final comparison of low- and high-power deposition at 500 °C. Similar optimization approaches have previously demonstrated that sputtering power and thermal treatment strongly influence the crystallinity, electrical transport, and optical properties of AZO thin films.^19,45^

**Figure 1:**
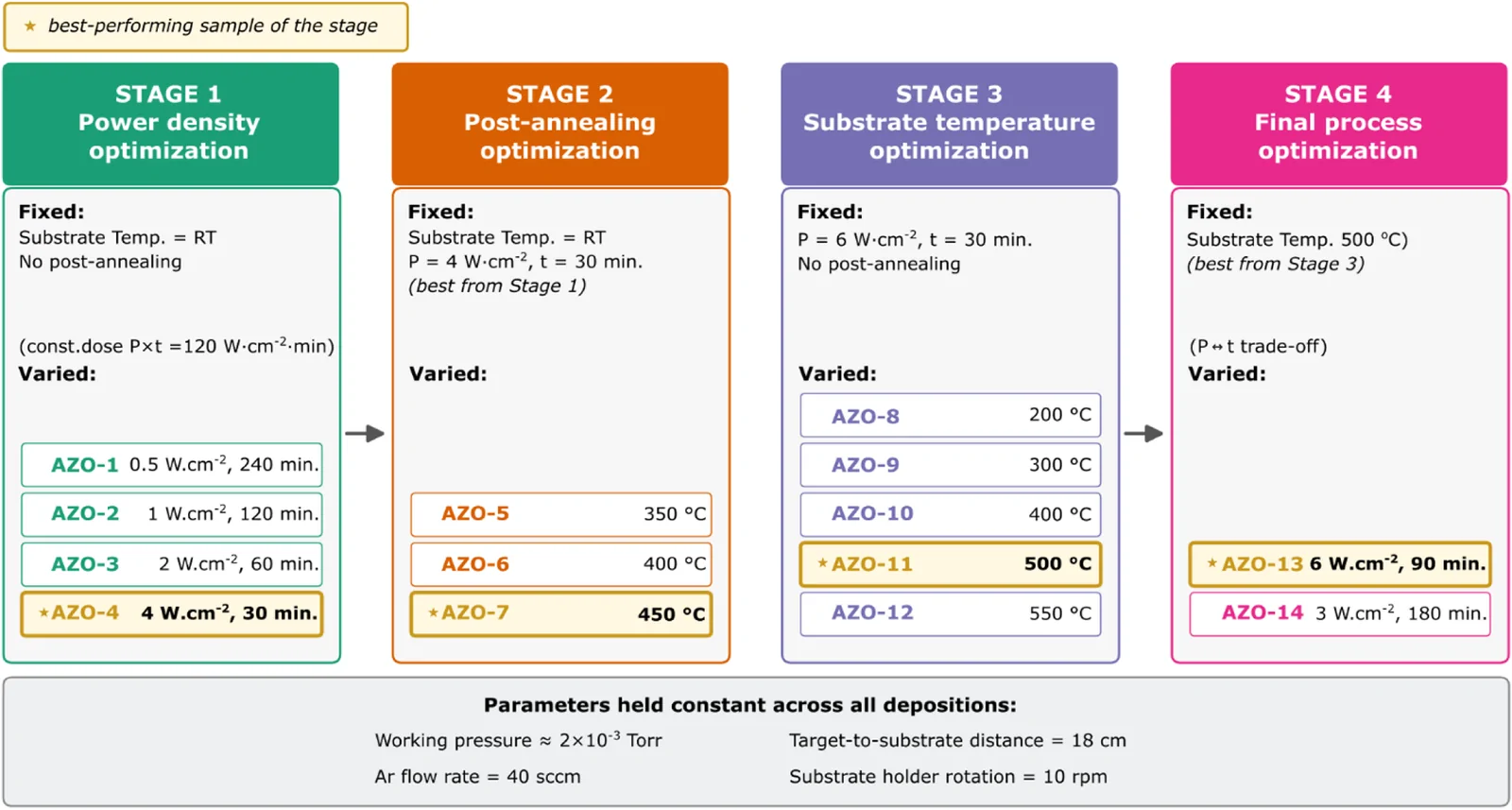
Schematic illustration of the sequential one-factor-at-a-time (OFAT) optimization strategy employed for AZO thin-film deposition. The optimum condition identified at each stage was fixed before proceeding to the next optimization step. Constant deposition parameters are summarized at the bottom of the figure, and the optimized sample at each stage is highlighted in yellow.

### Structural, Optical, and Electrical Properties of AZO Thin Films

The crystallographic properties of the AZO thin films were investigated by GIXRD to evaluate the effects of sputtering power, postdeposition annealing, substrate temperature, and deposition duration on crystal growth behavior. As shown in Figure 2a, all samples exhibited the characteristic diffraction peaks of hexagonal wurtzite ZnO, with the dominant reflection observed at 2θ ≈ 34.5–34.8°, corresponding to the ZnO (002) plane (Figure 2b). The strong (002) reflection indicates preferential *c*-axis-oriented growth perpendicular to the substrate surface, which is favorable for carrier transport in AZO-based transparent electrodes.^46,47^

**Figure 2:**
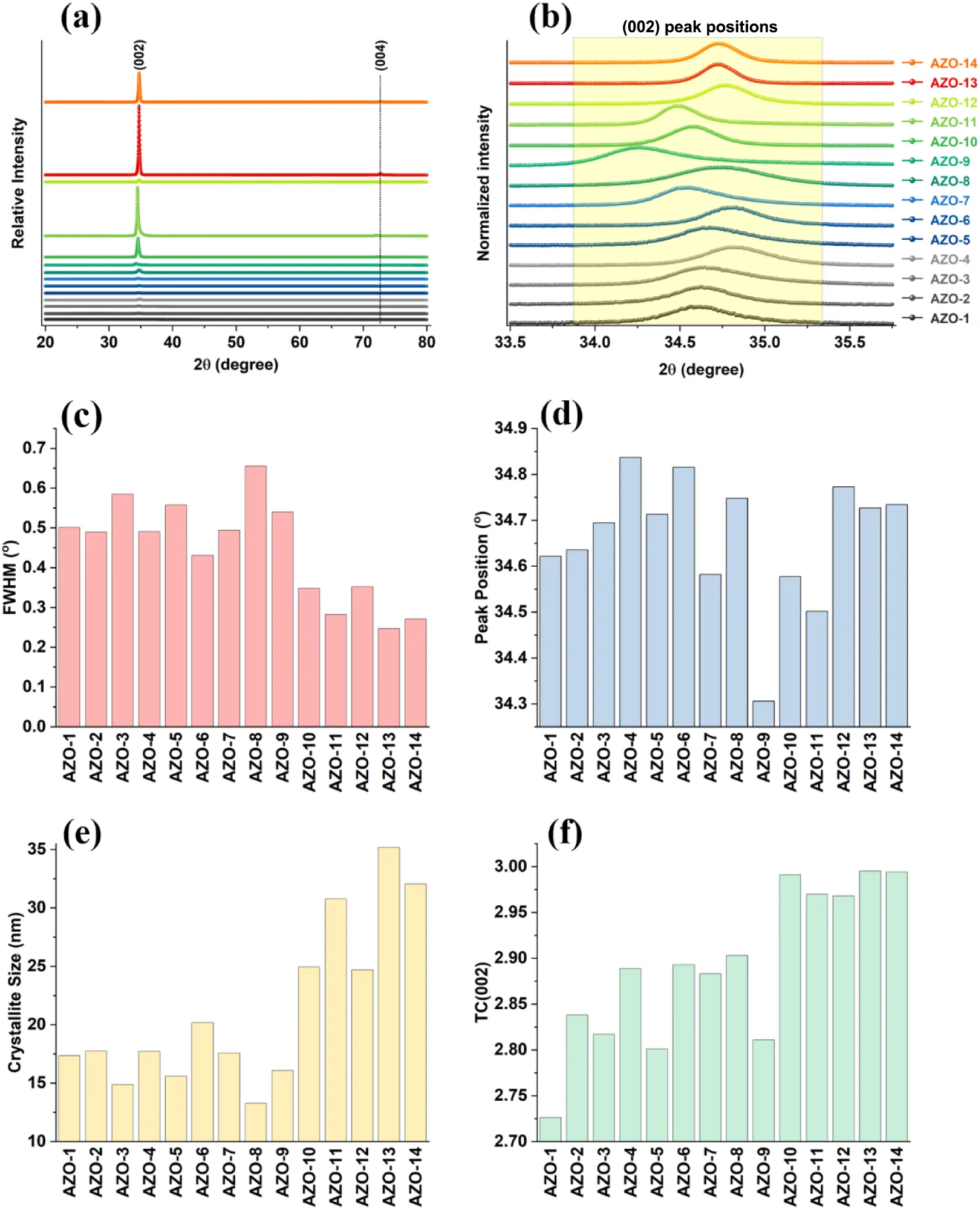
Structural characterization of AZO thin films: (a) XRD patterns of AZO-1–AZO-14, (b) enlarged ZnO (002) diffraction peak region, and variations of (c) fwhm, (d) peak position, (e) crystallite size, and (f) *TC­(002)* values.

The crystallite size (*D*), microstrain (ε), dislocation density (δ), lattice parameter (*c*), residual stress (σ), and texture coefficient (*TC*) were calculated from the XRD data to quantify the structural evolution of the films. The crystallite size was estimated to be using the Scherrer equation:

$$D=\frac{K\lambda }{\beta \cos\theta }$$

where *K* is the shape factor (0.9), λ is the X-ray wavelength, β is the full width at half-maximum (fwhm), and θ is the Bragg angle.^48^ The microstrain and dislocation density were calculated using

$$\varepsilon =\frac{\beta \cos\theta }{4}$$

and

$$\delta =\frac{1}{D^{2}}$$

respectively.^49,50^ The lattice parameter *c* and residual stress values were determined using Bragg’s law and the hexagonal wurtzite relation:

$$\frac{1}{d^{2}}=\frac{4}{3}\frac{h^{2}+hk+k^{2}}{a^{2}}+\frac{l^{2}}{c^{2}}$$

equation.^51^ The crystallographic texture was evaluated using the Harris texture coefficient method, where *TC­(002)* > 1 indicates preferential *c*-axis orientation.^52^ In addition, the relative (002) orientation fraction was estimated from

$$F_{\left(002\right)}=\frac{I_{\left(002\right)}}{I_{\left(100\right)}+I_{\left(002\right)}+I_{\left(101\right)}}$$

where *I*_(100)_, *I*_(002)_, and *I*_(101)_ are the diffraction peak intensities of the corresponding ZnO planes. The calculated structural parameters are provided in Table S2 (Supporting Information).

Figure 2c–f, plotted based on the data in Table S2 (Supporting Information), presents the variations in fwhm, peak position, crystallite size, and texture coefficient of the ZnO (002) diffraction peak as a function of the deposition conditions. The crystallite size calculated from the Scherrer equation increased significantly with increasing substrate temperature and optimized sputtering conditions, rising from 17.34 nm for AZO-1 to 35.20 nm for AZO-13, indicating enhanced grain growth and crystalline ordering. Simultaneously, the microstrain and dislocation density values decreased progressively, with the microstrain decreasing from 2.73 × 10^–3^ for AZO-8 to 1.03 × 10^–3^ for AZO-13 and the dislocation density decreasing from 5.68 × 10^15^ m^–2^ to 0.81 × 10^15^ m^–2^. These results indicate that elevated substrate temperatures and optimized growth conditions effectively suppress structural defects and lattice distortion, thereby reducing defect-assisted carrier scattering. The calculated lattice parameter values remained close to the standard ZnO lattice constant (*c*_0_ = 5.2066 Å), suggesting residual strain induced by Al incorporation and sputtering-related stress. Most samples exhibited compressive strain behavior, whereas AZO-9 showed a slight tensile deviation. The pronounced shift of the ZnO (002) peak toward lower 2θ values for AZO-9 suggests tensile lattice strain and local lattice distortion induced by the transition to substrate-heated growth conditions. Since this behavior is also accompanied by changes in crystallite size, microstrain, and residual stress parameters, the peak shift is considered to originate primarily from strain-related structural evolution rather than instrumental effects. In addition, the residual stress values generally decreased for highly crystalline films, particularly for AZO-10 and AZO-11, indicating partial strain relaxation during high-temperature growth. The texture coefficient *TC­(002)*, calculated using the Harris method, was greater than unity for all samples, confirming strong preferential *c*-axis orientation. The *TC­(002)* value increased systematically with optimized deposition conditions and reached a maximum value of approximately 2.995 for AZO-13. Similarly, the relative (002) orientation fraction approached unity for AZO-13 and AZO-14, indicating highly oriented columnar crystal growth promoted by enhanced surface adatom mobility during high-temperature sputtering.

Our optimized AZO-13 film exhibited a crystallite size of 35.20 nm together with a *TC­(002)* value of 2.995, indicating a highly oriented *c*-axis growth. The crystallite size is higher than, while the *TC­(002)* value is comparable to, those reported for recently developed sputtered AZO thin films prepared under optimized deposition conditions, where crystallite sizes of approximately 15–21 nm and *TC­(002)* values around 3.152 have been achieved. This comparison confirms that the optimized sputtering strategy employed in the present work yields structural quality comparable to current state-of-the-art AZO transparent electrodes.^53,54^

The Raman spectra of the AZO thin films shown in Figure 3a exhibited the characteristic vibrational features of hexagonal wurtzite ZnO, confirming successful crystalline AZO formation under all deposition conditions. The dominant Raman response observed in the 430–600 cm^–1^ region is associated with ZnO-related vibrational modes. In particular, the broad feature centered near ∼437–460 cm^–1^ corresponds to the nonpolar $E_{2}^{high}$ phonon mode originating from oxygen sublattice vibrations, which is commonly regarded as an indicator of crystalline quality and long-range structural ordering in ZnO systems. Additional Raman bands observed near ∼275–282 cm^–1^, ∼515–525 cm^–1^, and ∼560–590 cm^–1^ are consistent with anomalous Raman modes previously reported for rf-sputtered AZO thin films.^55–57^ Although these additional Raman bands provide indirect evidence of the structural modifications induced by Al doping, they should not be considered direct proof of Al substitution into the ZnO lattice. Instead, they reflect the evolution of defect-related vibrational modes accompanying Al incorporation and sputtering-induced lattice disorder. The modes near ∼275 and ∼515 cm^–1^ are generally attributed to defect-activated silent $B_{1}^{low}$ and $B_{1}^{high}$ -related phonons associated with Al incorporation, lattice disorder, and relaxation of Raman selection rules,^58^ while the broad band near ∼580 cm^–1^ corresponds to overlapping A_1_(LO) and/or E_1_(LO) modes associated with oxygen-vacancy-related defects, impurity scattering, and carrier–phonon interactions in AZO films.^55,56^

**Figure 3:**
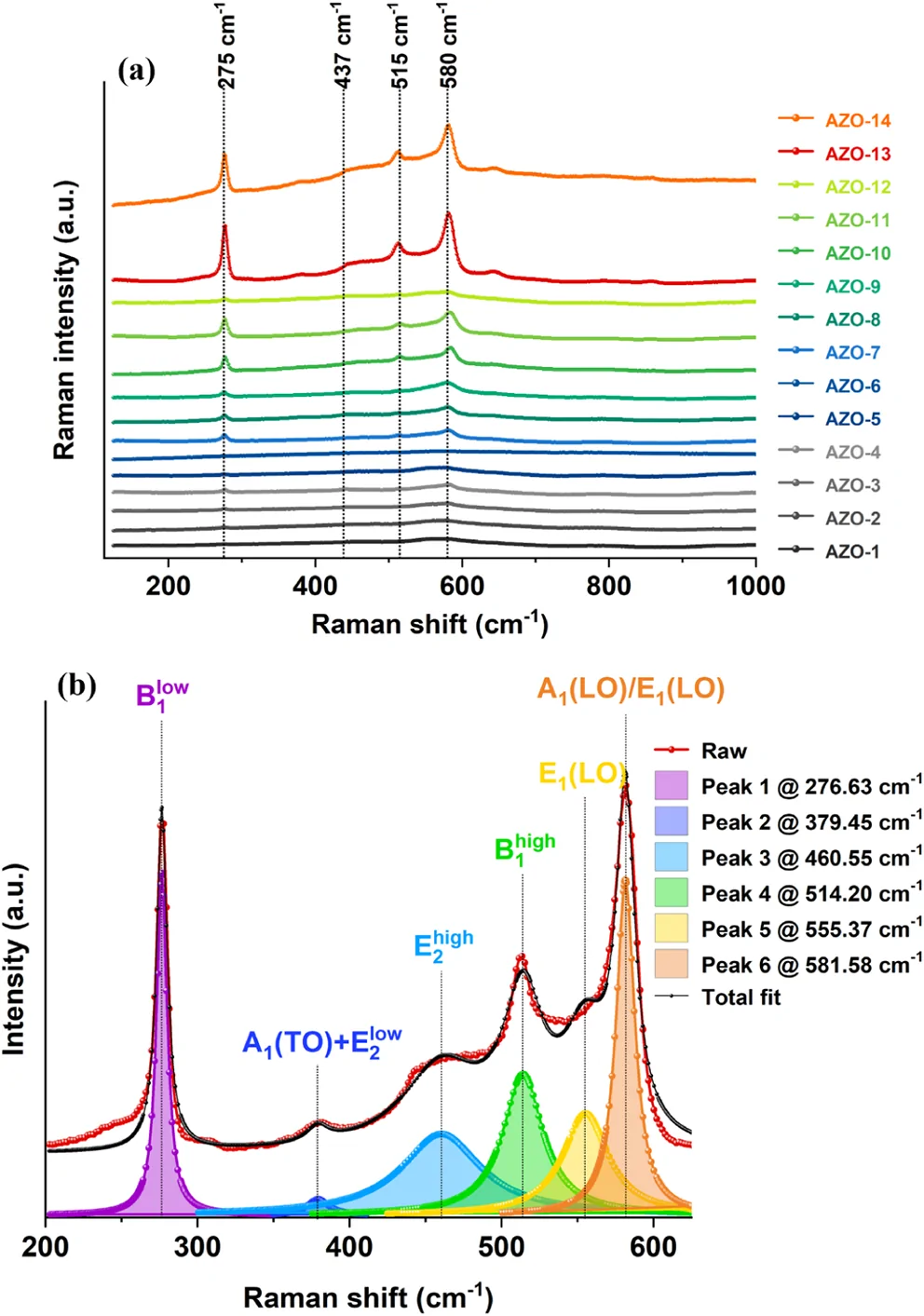
(a) Raman characterization of AZO thin films deposited under different sputtering conditions and (b) Lorentzian deconvolution analysis of the Raman spectrum of the optimized AZO-13 film.

A systematic evolution in Raman intensity and line width was observed with increasing substrate temperature and optimized sputtering conditions. The room-temperature-grown samples exhibited relatively broad and weak Raman bands, indicating higher structural disorder and defect density, whereas postdeposition annealing partially improved crystalline ordering. The most significant enhancement was observed for the high-temperature-grown films, particularly AZO-11 and AZO-13, where the Raman peaks exhibited higher intensity and narrower line widths. In addition, the I_580_/I_515_ ratio increased from 1.42 for AZO-1 to 3.47 for AZO-13, consistent with the enhanced crystallinity and stronger preferential (002) orientation observed in XRD analysis. Taken together, the Raman results confirm that an elevated substrate temperature and optimized sputtering conditions significantly improve the structural ordering and vibrational properties of AZO thin films.

To further investigate the vibrational behavior of the optimized film, Lorentzian deconvolution analysis was performed for the Raman spectrum of AZO-13, as shown in Figure 3b. The deconvoluted spectrum revealed six vibrational contributions centered at approximately 276.63, 379.45, 460.55, 514.20, 555.37, and 581.58 cm^–1^. The peaks near ∼276.63 and ∼514.20 cm^–1^ are attributed to defect-activated anomalous $B_{1}^{low}$ and $B_{1}^{high}$ -related phonon modes associated with Al incorporation and lattice disorder.^58^ The broad feature near ∼460.55 cm^–1^ corresponds to the $E_{2}^{high}$ phonon mode associated with oxygen sublattice vibrations and crystalline ordering, while the contributions near ∼555.37 and ∼581.58 cm^–1^ originate from defect-related *LO* phonon scattering associated with oxygen vacancies, donor-type defects, and carrier–phonon interactions. In particular, the intense peak near ∼581.58 cm^–1^ corresponds to overlapping *A*_1_ *(LO)* and *E*_1_ *(LO)* modes characteristic of doped ZnO systems.^56^ These results collectively confirm the coexistence of crystalline wurtzite ZnO ordering and defect-assisted vibrational scattering associated with Al incorporation and sputtering-related lattice disorder.

The optical transmittance spectra shown in Figure 4a–d demonstrate that the sputtering conditions strongly influence the optical quality of the AZO thin films. All samples exhibited relatively high transparency in the visible region, confirming their suitability for photovoltaic applications. Previous studies have similarly reported that the optical transparency and electrical performance of sputtered AZO films strongly depend on deposition parameters such as sputtering power, substrate temperature, and film thickness.^19,59^ As summarized in Figure 4e, the room-temperature-grown films (AZO-1–AZO-4) exhibited average visible transmittance values between ∼62.6% and ∼77.4%, where increasing sputtering power resulted in reduced transparency due to enhanced optical scattering and defect formation. Following postdeposition annealing (AZO-5–AZO-7), the average visible transmittance increased from 73.5% to 78.3%, indicating improved structural ordering and reduced defect-related optical losses.^60^ A more pronounced enhancement was observed for the substrate-heated films (AZO-8–AZO-12), where the average visible transmittance exceeded 78% and reached ∼83.6% for AZO-12. This behavior is consistent with the improved crystalline ordering and reduced lattice disorder observed in the XRD and Raman analyses.^61,62^ Although reflectance measurements were not performed, the enhanced transparency of the high-temperature-grown films may be associated with reduced defect-assisted optical losses and suppressed grain-boundary scattering.^63,64^ The final optimization stage shown in Figure 4d revealed that AZO-13 exhibited the highest average visible transmittance (∼86.0%), whereas AZO-14 showed a lower value (∼74.8%), indicating that optimized sputtering conditions and deposition duration significantly reduce optical scattering losses. The relationship between average visible transmittance and optical bandgap obtained from Tauc plot analysis is presented in Figure 4f. In general, films with higher transparency also exhibited relatively wider optical bandgaps, suggesting reduced sub-bandgap absorption associated with defect states. Among all samples, AZO-13 exhibited the most favorable balance between optical transparency and bandgap energy.

**Figure 4:**
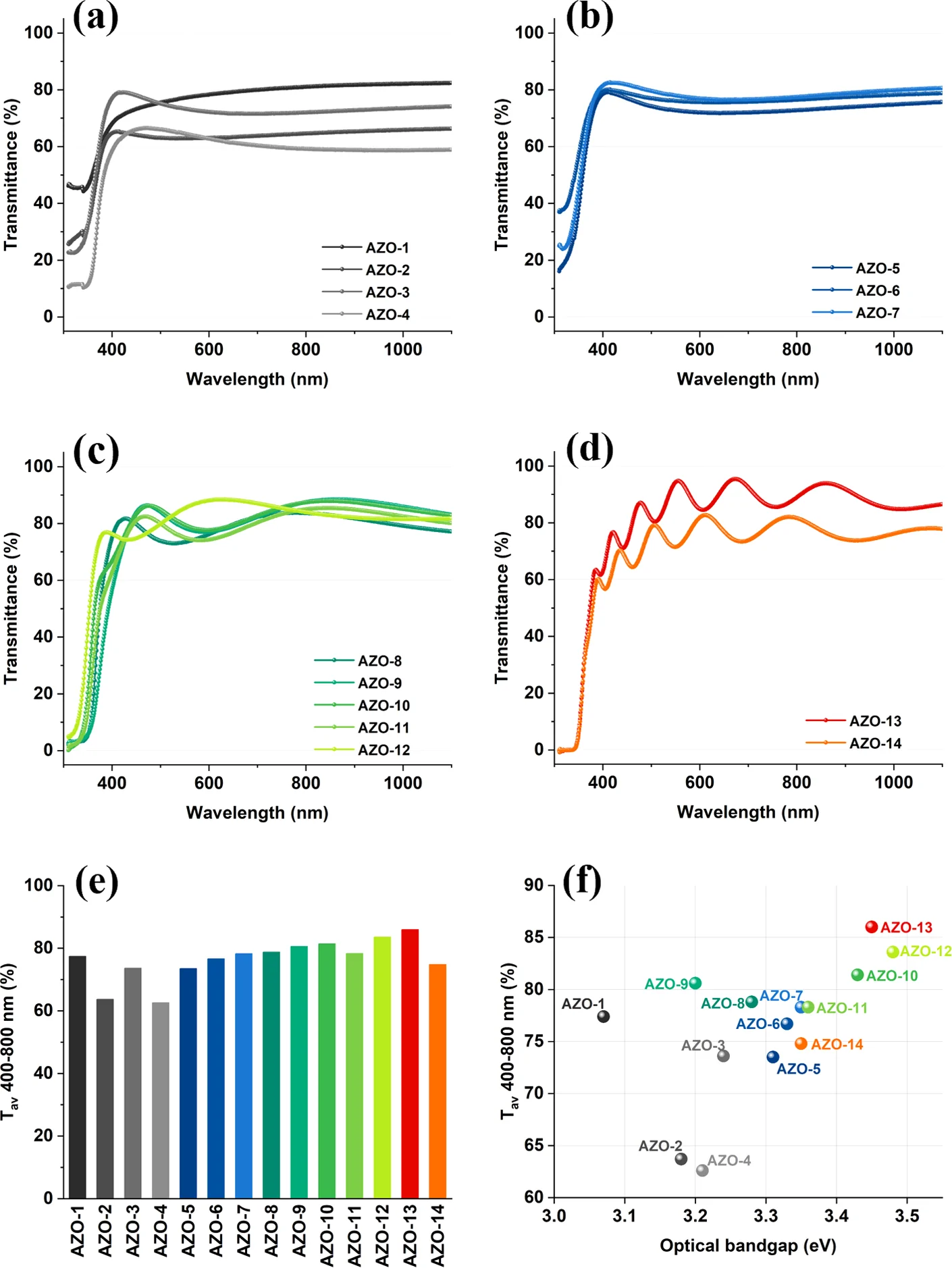
Optical characterization of AZO thin films: transmittance spectra of (a) AZO-1–AZO-4, (b) AZO-5–AZO-7, (c) AZO-8–AZO-12, and (d) AZO-13/AZO-14 films, together with (e) average visible transmittance values (400–800 nm) and (f) the relationship between average transmittance and optical bandgap energy.

The optimized AZO-13 film exhibited an average visible transmittance of approximately 86.0% and an optical bandgap of 3.45 eV, consistent with recently reported sputtered AZO thin films exhibiting transmittance values around 85% and optical bandgaps between 3.20 and 3.60 eV.^32,65^ These results confirm the competitive optical performance achieved through optimized sputtering conditions.

Electrical characterization revealed significant improvements in the conductivity of the AZO thin films with systematic process optimization. The resistivity (ρ) and electrical conductivity (σ) values were calculated using

$$\rho =R_{S}\times t$$

and

$$\sigma =\frac{1}{\rho }$$

where R_S_ is the sheet resistance obtained from the two-electrode I–V measurements described above and *t* is the film thickness measured by optical profilometry (Figure S2). Since the conductivity values were estimated from thickness and sheet resistance data, the reported values should be interpreted within the limitations of the applied measurement geometry.

As summarized in Figure 5a–d and Table S3 (Supporting Information), the electrical conductivity increased from 5.98 × 10^–2^ S·cm^–1^ for AZO-1 to 9.85 × 10^2^ S·cm^–1^ for AZO-13. This improvement strongly correlates with the XRD and Raman results, which revealed larger crystallite size, reduced microstrain, lower dislocation density, and enhanced preferential *c*-axis orientation for the optimized films. The sheet resistance values shown in Figure 5a decreased systematically with process optimization, while the calculated conductivity values in Figure 5b exhibited the opposite trend. In the room-temperature-grown series, increasing sputtering power improved electrical conductivity but also promoted optical losses and structural disorder. Postdeposition annealing partially restored crystalline ordering and reduced defect-related carrier scattering, leading to lower sheet resistance and improved conductivity. A more pronounced enhancement was achieved by substrate heating, which promoted improved crystallinity and reduced lattice disorder, consistent with the XRD and Raman analyses.

**Figure 5:**
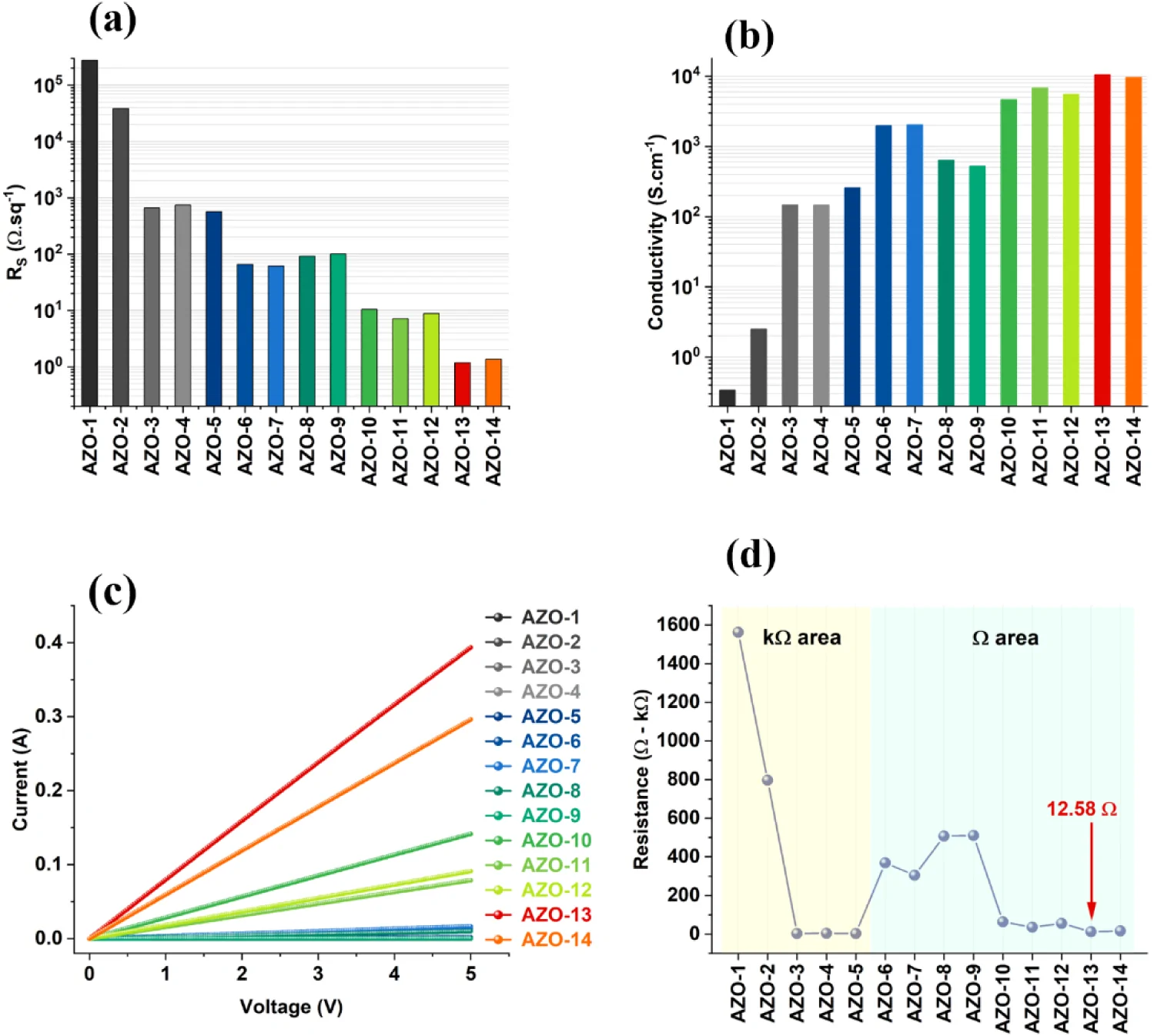
Electrical characterization of AZO thin films: (a) sheet resistance, (b) electrical conductivity, (c) I–V characteristics, and (d) resistance evolution during the optimization process.

The current–voltage characteristics shown in Figure 5c and d, obtained from the measurements presented in Figure S3 using the Ag contact geometry shown in Figure S4, further demonstrate the progressive improvement in charge transport behavior with increasing substrate temperature and process optimization. The I–V curves were obtained between two 5 mm long Ag electrodes separated by 5 mm under applied voltages between 0 and 5 V. The low-temperature-grown samples exhibited weak current response, whereas AZO-13 and AZO-14 showed significantly enhanced current conduction with nearly linear ohmic behavior. The resistance evolution summarized in Figure 5d indicates a transition from the kΩ region to the Ω region after substrate heating and final optimization. Among all samples, AZO-13 exhibited the lowest resistance (∼12.58 Ω), the lowest sheet resistance (1.26 × 10^1^ Ω·sq^–1^), and the highest calculated electrical conductivity (∼9.85 × 10^2^ S·cm^–1^). Although the reduced sheet resistance is partially attributable to the increased film thickness obtained during the final optimization stage, the electrical resistivity, which is independent of film thickness, also exhibited the lowest value for AZO-13 (1.01 × 10^–3^ Ω·cm). This result indicates that the improved electrical performance cannot be attributed solely to thickness but also reflects the enhanced intrinsic electrical quality arising from improved crystallinity, stronger preferential (002) orientation, and reduced lattice disorder. Consequently, the selection of AZO-13 as the optimum transparent electrode was based on the combined evaluation of its structural, optical, and intrinsic electrical properties rather than on sheet resistance alone. Compared with AZO-14, the improved optoelectronic performance of AZO-13 indicates that both deposition duration and sputtering conditions play critical roles in balancing electrical conductivity and optical transparency. It should be noted that the thickness of AZO-13 was intentionally chosen to match the typical thickness of commercially available AZO-coated glass substrates (∼800–900 nm). Accordingly, the final comparison was performed under practically relevant electrode thicknesses, and the superior performance of AZO-13 should be attributed to the combined effects of optimized growth conditions and electrode thickness rather than thickness alone.

To further verify the transport characteristics discussed above, Hall effect measurements were performed on representative AZO films corresponding to different stages of the optimization process together with the commercial FTO substrate. The measured Hall parameters are summarized in Table S1 (Supporting Information). All investigated samples exhibited n-type conductivity. The carrier concentration increased markedly from AZO-1 to AZO-11 and remained on the order of 10^21^ cm^–3^ for the optimized AZO-13 film. Although AZO-14 exhibited considerably higher carrier mobility, its carrier concentration decreased substantially. Nevertheless, the enhanced mobility compensated for this reduction, resulting in a comparable electrical conductivity relative to AZO-13. These results indicate that the conductivity enhancement observed during the optimization process is governed primarily by the increase in carrier concentration while maintaining sufficient carrier mobility, rather than by mobility enhancement alone. Furthermore, the Hall-derived transport parameters are in good agreement with the conductivity trends obtained from the sheet resistance measurements, thereby providing direct experimental support for the electrical transport discussion presented above.

A comparison between the Hall-derived carrier concentration and the optical bandgap for the five representative samples (AZO-1, AZO-7, AZO-11, AZO-13, and AZO-14) reveals an approximate positive trend: *E*_*g*_ increases monotonically from 3.07 to 3.45 eV as the carrier concentration increases from 1.13 × 10^20^ to 2.12 × 10^21^ cm^–3^ across AZO-1, AZO-14, AZO-7, and AZO-13. This behavior is qualitatively consistent with a Burstein–Moss-type shift of the apparent optical bandgap with increasing free-carrier density,^66,67^ as similarly reported for Al-doped ZnO films.^68^ However, AZO-11, which exhibits the highest carrier concentration among the investigated samples (2.45 × 10^21^ cm^–3^), shows a slightly lower bandgap (3.36 eV) than AZO-13, indicating that the trend is not strictly monotonic across the entire series, consistent with previous reports of competing Burstein–Moss widening and bandgap-renormalization effects in heavily doped AZO films at high carrier concentrations.^68^ This deviation coincides with a marginally smaller crystallite size and higher microstrain for AZO-11 relative to AZO-13 (Table S2), suggesting that structural disorder may also contribute to the observed bandgap variations alongside the carrier-concentration-related effect. Accordingly, while an approximate carrier-concentration-dependent trend is observed, the optical bandgap variations across the series likely reflect a combined influence of free-carrier density and structural disorder rather than a single dominant mechanism.

To further assess the overall optoelectronic performance of the transparent electrodes, the Haacke high-resolution figure of merit (FOM­(H-HR)) was calculated using the average visible transmittance and sheet resistance values (Supporting Information Table S4).^69–71^ The FOM­(H-HR) increased progressively with the optimization of the sputtering parameters and reached a maximum value of 0.668 for AZO-13, corresponding to an approximately 0.6% improvement over the commercial FTO substrate (0.664). This result demonstrates that the optimized AZO-13 film achieved an excellent balance between optical transparency and electrical conductivity, confirming its suitability as a transparent conductive electrode.

These combined structural, optical, and electrical improvements establish AZO-13 as the most promising candidate among the films investigated for the integration of PSCs.

The electrical properties of the optimized AZO-13 film are comparable to those reported for recently developed sputtered AZO transparent electrodes. The low sheet resistance and high electrical conductivity achieved in this study confirm the effectiveness of the proposed optimization strategy.^22,32^

To further clarify whether the optimized sputtering conditions also modified the surface chemical environment of the AZO films, XPS measurements were performed on the reference AZO-1 and the optimized AZO-13 samples. The corresponding survey spectrum (Figure S5) confirms that both films consist exclusively of Zn, Al, and O, together with the adventitious C 1s signal used for charge correction, indicating the absence of detectable impurity phases.

The high-resolution Zn 2p spectra of AZO-1 and AZO-13 are presented in Figure 6a, while the fitted binding energies are summarized in Table S5. The Zn 2p spectra exhibit the characteristic spin–orbit doublet with Zn 2p_3/2_ located at 1021.27 eV for AZO-1 and 1021.39 eV for AZO-13, whereas the corresponding Zn 2p_1/2_ peaks appear at 1044.37 and 1044.59 eV, respectively. The spin–orbit splitting remains approximately 23.1–23.2 eV for both films, which is characteristic of Zn^2+^ occupying tetrahedral sites in the wurtzite ZnO lattice.^72^ No additional components associated with metallic Zn or secondary zinc-containing phases are detected, demonstrating that optimization of the sputtering parameters did not alter the oxidation state of zinc but preserved the ZnO crystal framework. The fitted peak parameters, including binding energy, fwhm, and peak area, are listed in Table S5. These observations are fully consistent with recent XPS studies on sputtered and ALD-grown AZO films, where Zn 2p binding energies near 1021–1022 eV are assigned to Zn^2+^ bonded with lattice oxygen.^72,73^

**Figure 6:**
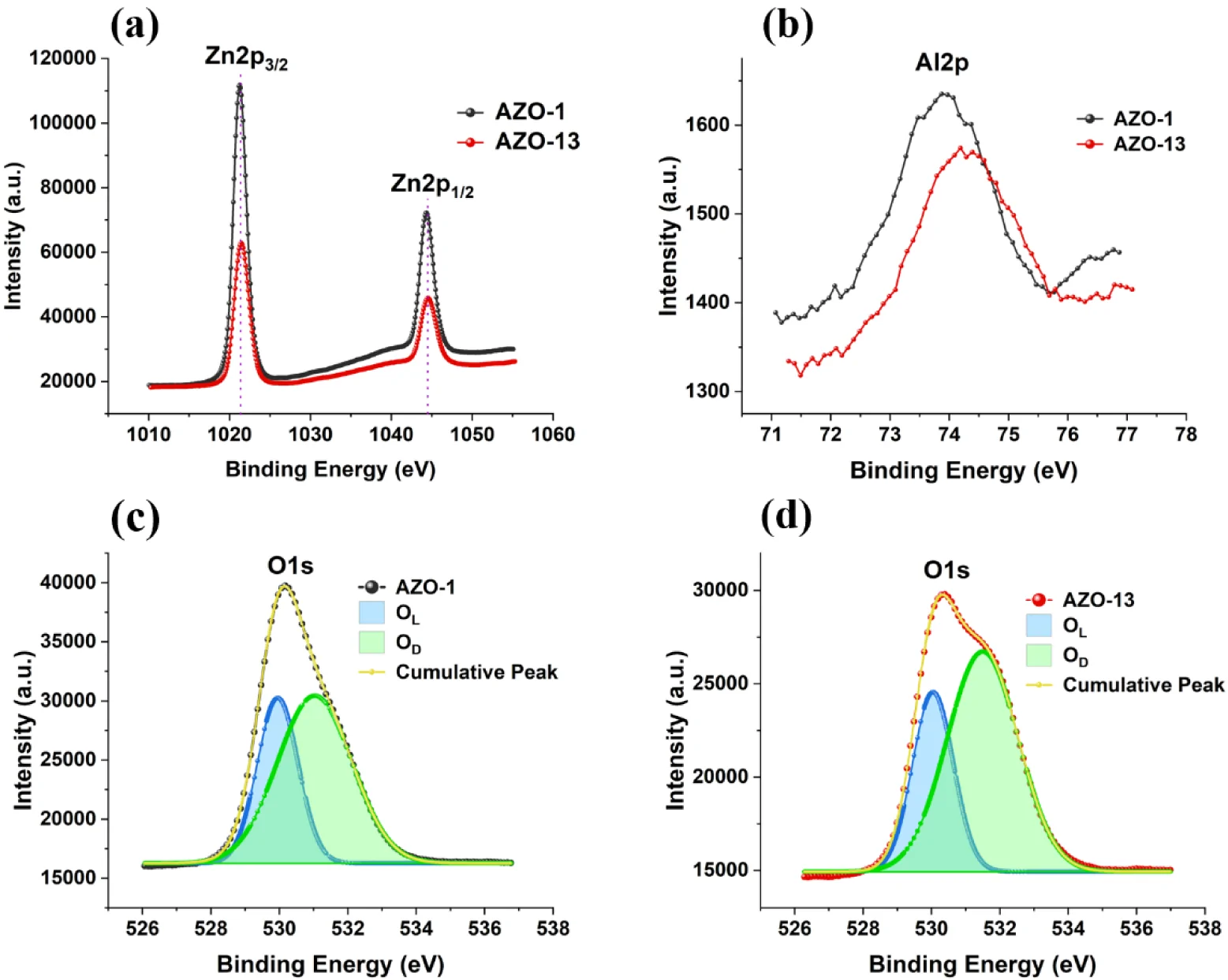
High-resolution XPS spectra of the AZO-1 (reference) and AZO-13 (optimized) thin films: (a) Zn 2p, (b) Al 2p, and O 1s deconvolution spectra of (c) AZO-1 and (d) AZO-13, showing the lattice oxygen (O_L_) and oxygen-deficient (O_D_) components.

The Al 2p spectra shown in Figure 6b exhibit a single symmetric peak centered at 73.86 eV for AZO-1 and 74.22 eV for AZO-13, confirming that aluminum is predominantly incorporated as Al^3+^ within the ZnO lattice rather than forming separate Al-rich oxide phases. The modest positive shift of the Al 2p binding energy from 73.86 to 74.22 eV is consistent with a strengthening of the Al–O bonding character upon optimization, reflecting a more complete and symmetric oxide coordination of Al^3+^ within the improved ZnO lattice rather than a change in oxidation state. Similar slight increases in the Al 2p binding energy have been reported for optimized Al-doped ZnO-based transparent conducting films and were likewise attributed to enhanced Al–O bond ordering rather than to the formation of secondary aluminum oxide phases.^74^ The absence of additional Al 2p components indicates that the optimized deposition conditions promote substitutional incorporation of Al into Zn lattice sites without detectable phase segregation. Such substitutional Al is responsible for the generation of additional free electrons and therefore contributes directly to the enhanced electrical conductivity of the optimized AZO-13 film. This interpretation agrees well with the electrical measurements, where the carrier concentration increases from 1.13 × 10^20^ to 2.12 × 10^21^ cm^–3^ while the sheet resistance decreases dramatically from 1.85 × 10^4^ to 2.29 × 10^1^ Ω·sq^–1^ after optimization. Similar behavior has been widely reported for high-quality AZO transparent electrodes in which substitutional Al acts as an efficient shallow donor without inducing secondary phases.^75^

The O 1s spectra were further analyzed by deconvolution into two components, as shown in Figure 6c and 6d. The lower binding-energy component located at approximately 530.0 eV is assigned to lattice oxygen (O_L_) associated with Zn–O bonds in the wurtzite structure, whereas the higher binding-energy component centered at approximately 531.0–531.5 eV corresponds to oxygen-deficient regions (O_D_) related to oxygen-vacancy-associated oxygen species.^76^ The relative contribution of the O_D_ component increases from 64.86% in AZO-1 to 68.10% in AZO-13, while the O_L_ contribution decreases slightly from 35.14% to 31.90%. This result indicates that optimization of the sputtering parameters promoted the formation of oxygen-deficient regions, which are known to increase the free-electron concentration in n-type ZnO-based transparent conducting oxides. The XPS results, therefore, correlate remarkably well with the Hall-effect measurements, where the carrier concentration increases by more than 1 order of magnitude together with a significant reduction in resistivity from 5.56 × 10^–2^ to 1.85 × 10^–3^ Ω·cm and an increase in conductivity to approximately 5.4 × 10^2^ S·cm^–1^. The simultaneous increase in carrier concentration, reduction in resistivity, and enhancement of the O_D_ contribution are consistent with the optimized sputtering conditions having generated an appropriate concentration of electrically active oxygen-deficiency-related defects while maintaining the ZnO lattice integrity. Similar correlations between oxygen-defect-related O 1s components and the electrical performance of AZO transparent electrodes have recently been reported for both thin-film electronics and optoelectronic applications.^77^

Overall, the XPS analysis demonstrates that the optimized AZO-13 film preserves the Zn^2+^ and Al^3+^ chemical states characteristic of high-quality AZO while exhibiting a slightly higher concentration of oxygen deficiency-related species. Combined with the improved crystallinity, higher average visible transmittance, significantly lower sheet resistance, and superior Haacke figure of merit discussed above, these results provide direct evidence that AZO-13 possesses the most favorable combination of structural, electrical, and surface chemical properties among the investigated films. Accordingly, AZO-13 was selected as the optimized transparent conducting electrode for subsequent perovskite solar cell fabrication and direct comparison with the commercial FTO substrate.

### Comparison of FTO and Optimized AZO-13 Electrodes for PSC Applications

The optimized AZO-13 film was directly compared with commercial FTO to evaluate its applicability as an alternative TCO for PSC fabrication. Figure 7 presents the comparative structural and optical characterization of both electrodes prior to the PSC integration. As shown in Figure 7a, FTO exhibited the characteristic diffraction peaks of tetragonal SnO_2_, whereas AZO-13 displayed the typical reflections of hexagonal wurtzite ZnO with a dominant (002) preferential orientation, indicating a highly oriented *c*-axis growth. The Raman spectra shown in Figure 7b further confirm the distinct vibrational characteristics of the two TCO materials. FTO exhibited relatively weak SnO_2_-related Raman features, while AZO-13 showed intense ZnO-related and defect-assisted phonon modes characteristic of the crystalline AZO films.

**Figure 7:**
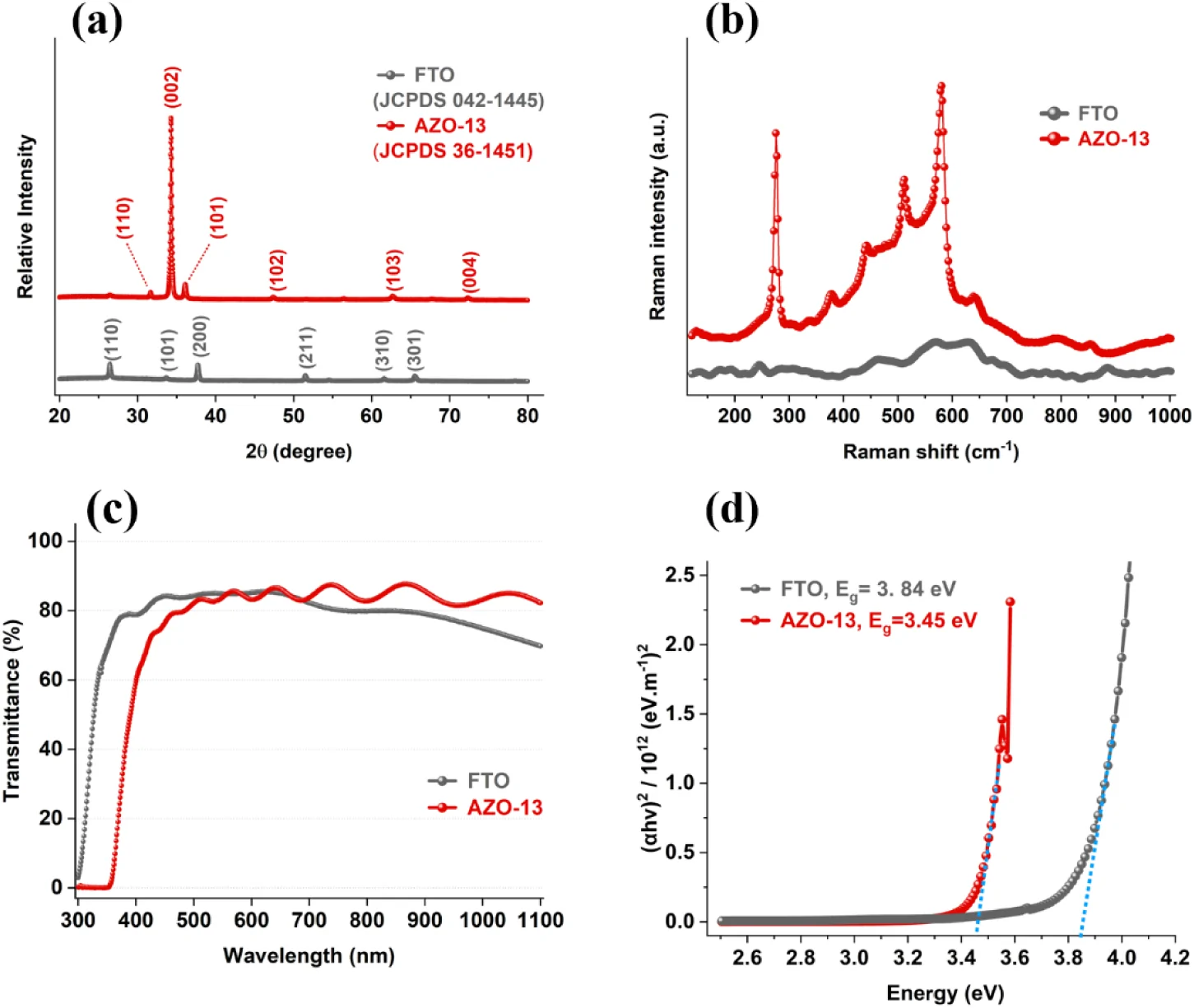
Comparative structural and optical characterization of commercial FTO and optimized AZO-13. (a) XRD patterns, (b) Raman spectra, (c) optical transmittance spectra, and (d) Tauc plots used for optical bandgap determination.

The optical transmittance spectra presented in Figure 7c revealed that both electrodes possess high visible transparency suitable for photovoltaic applications, although AZO-13 exhibited a slightly higher transmittance over a broad wavelength range. The Tauc plots shown in Figure 7d yielded optical bandgap values of approximately 3.84 eV for FTO and 3.45 eV for AZO-13, reflecting the different electronic structures of SnO_2_- and ZnO-based TCOs. In summary, Figure 7 highlights the structural and optical differences between commercial FTO and optimized AZO-13 electrodes prior to the PSC fabrication. The film thickness value and optical profilometry image used for the Tauc plot analysis of AZO-13 are provided in Figure S2 (Supporting Information).

Although commercial FTO exhibits higher transmittance in the UV region, AZO-13 shows improved transparency at longer wavelengths, particularly toward the near-infrared region. This behavior is important for tandem photovoltaic architectures, especially perovskite/Si tandem solar cells, where the semitransparent perovskite top cell must efficiently transmit long-wavelength photons to the bottom Si subcell. Recent studies have emphasized that parasitic absorption and optical losses originating from transparent conductive electrodes directly influence current matching and tandem efficiency in next-generation tandem devices.^27,31^ In this context, the enhanced near-infrared transparency of AZO-based electrodes may provide advantages for tandem-oriented photovoltaic applications despite their relatively lower UV transmittance compared with FTO. Furthermore, indium-free AZO electrodes have recently attracted increasing attention as low-cost and scalable alternatives for tandem solar cells due to their favorable optoelectronic transparency in the visible–near-infrared region and compatibility with large-area fabrication processes.^30,78^

Figure 8 presents the comparative surface morphology and topographical characteristics of commercial FTO and optimized AZO-13 thin films. The FESEM images shown in Figure 8a and Figure 8b reveal that both electrodes possess continuous polycrystalline surface structures with noticeable differences in grain morphology and surface compactness. The FTO exhibits an irregular grain distribution and densely packed granular features with relatively rough morphology. In contrast, AZO-13 shows a more homogeneous grain distribution with well-defined grain boundaries and comparatively uniform surface coverage. The enlarged FESEM image further reveals larger and more distinguishable grains for AZO-13, consistent with the enhanced crystallite growth observed in the XRD analysis.

**Figure 8:**
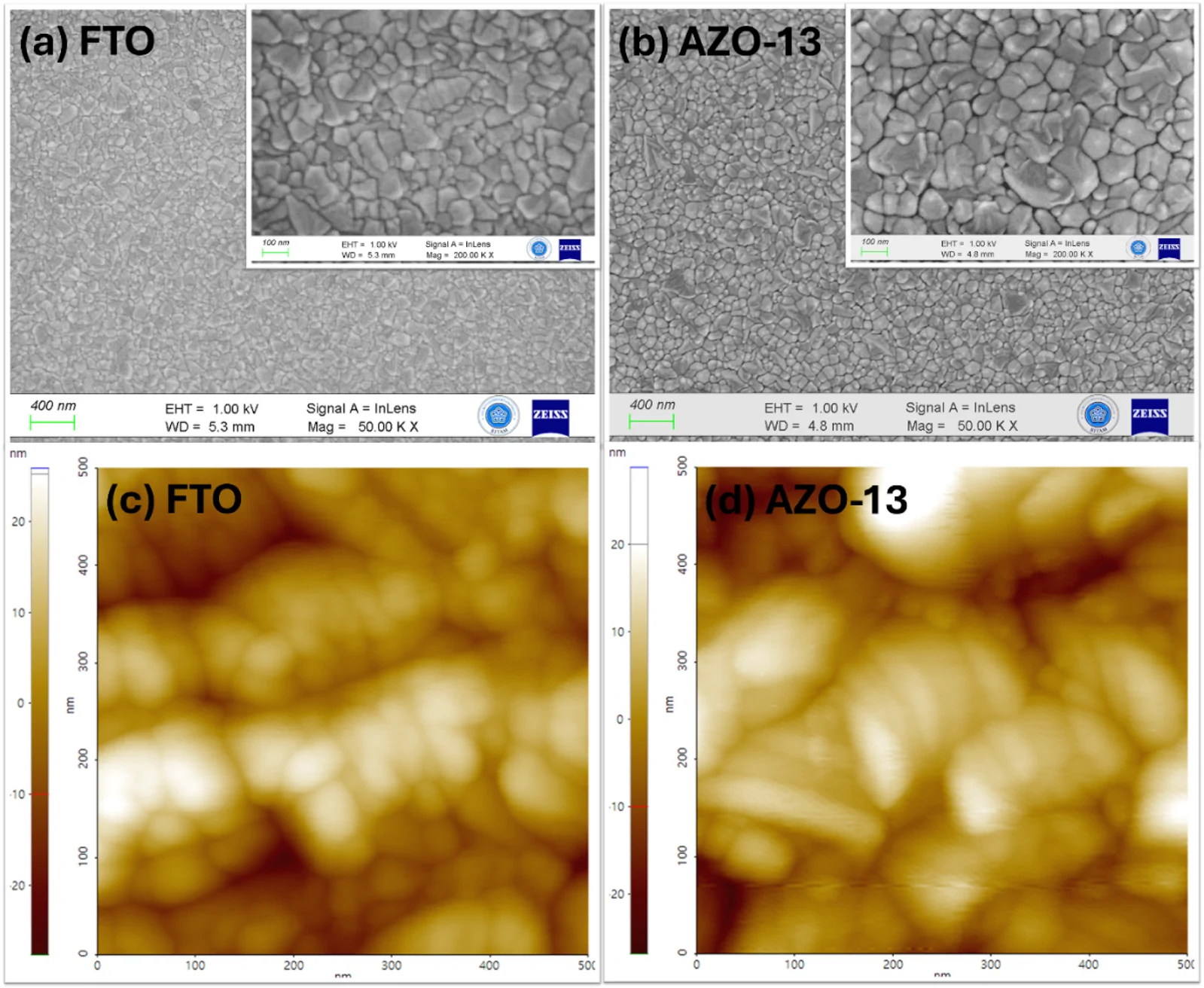
Comparative FESEM and AFM surface morphology of commercial FTO and optimized AZO-13 electrodes. (a) FESEM images of the FTO substrate and (b) optimized AZO-13 thin film recorded at magnifications of 50 kX and 200 kX. (c) AFM topography image of the FTO substrate and (d) AZO-13 thin film measured over a 0.5 μm × 0.5 μm scan area.

The AFM images presented in Figure 8c and Figure 8d further confirm the morphological differences between the two electrodes. FTO exhibits pronounced surface fluctuations and sharper topographical features, indicating a rougher surface morphology, whereas AZO-13 appears to have a more uniform surface morphology. This morphology may be beneficial for the subsequent ETL and perovskite deposition. Together, the FESEM and AFM analyses indicate that the optimized AZO-13 film possesses a compact and comparatively smoother surface morphology that is suitable for PSC integration. Quantitative AFM analysis further confirms this observation, yielding an RMS roughness (Rq) of 8.753 nm and an average roughness (Ra) of 7.087 nm for AZO-13, compared with 9.375 and 7.537 nm for the commercial FTO substrate, respectively, indicating that the optimized AZO film possesses a slightly smoother surface, as summarized in Table S6 (Supporting Information).

### Charge Dynamics of Perovskite Films on FTO and AZO-13

Figure 9 presents the structural and optoelectronic comparison of perovskite layers deposited on commercial FTO and optimized AZO-13 transparent conductive electrodes coated with compact TiO_2_ (c-TiO_2_) layers. Since all subsequent PSC fabrication steps were identical, the observed differences mainly originate from the underlying TCO properties. The XRD patterns shown in Figure 9a confirm the successful formation of crystalline perovskite phases on both substrates. The diffraction peaks marked by blue triangles correspond to the characteristic reflections of the perovskite structure. Compared with perovskite/FTO, the perovskite/AZO-13 film exhibits relatively strong and sharper diffraction peaks, particularly near ∼14° and ∼28°, indicating enhanced crystallinity and preferential crystal growth. Similar substrate-dependent crystallization effects have previously been reported for engineered oxide interfaces in PSCs.^79^ It is also worth noting that the optimized AZO-13 film was deposited at 500 °C, which is higher than the subsequent 450 °C temperature employed during c-TiO2 deposition. Moreover, the characteristic AZO diffraction peaks remain observable in Figure 9a, indicating that the crystalline AZO phase was preserved throughout the device fabrication process. The absorbance spectra shown in Figure 9b reveal strong visible-light absorption for both perovskite films with a sharp absorption edge near ∼760 nm. Although the perovskite/FTO sample exhibits slightly higher absorbance intensity, this behavior is likely associated with increased optical scattering caused by the rougher FTO surface morphology. Similar morphology-dependent optical responses have been reported for perovskite films deposited on different TCO architectures.^80,81^ The steady-state PL spectra shown in Figure 9c exhibit significantly stronger emission intensity for the perovskite/AZO-13 sample than for perovskite/FTO. In planar perovskite/c-TiO_2_/TCO structures, stronger PL emission generally indicates less efficient electron extraction from the perovskite absorber into the underlying electron-transport stack, resulting in enhanced radiative recombination within the perovskite layer. Accordingly, the higher PL intensity observed for perovskite/AZO-13 suggests less efficient interfacial charge extraction compared with the FTO-based structure. This interpretation is consistent with previous reports showing that variations in interfacial charge extraction at oxide/perovskite interfaces directly influence the steady-state PL intensity.^82,83^ The TRPL decay profiles shown in Figure 9d and the fitted lifetime parameters summarized in Table 2 provide further insight into the carrier decay dynamics. The decay curves were fitted using a biexponential model, where the fast component (τ_1_) is associated with rapid interfacial decay processes, while the slow component (τ_2_) reflects carrier recombination within the perovskite bulk. Compared with the FTO-based structure, the perovskite/AZO-13 sample exhibited a shorter average carrier lifetime (128 ns versus 199 ns). The TRPL decay therefore reflects the overall carrier recombination dynamics following photoexcitation rather than the efficiency of interfacial charge extraction alone. Accordingly, the steady-state PL and TRPL results probe different physical processes and should be interpreted separately. The stronger steady-state PL emission indicates less efficient electron extraction from the perovskite absorber into the underlying electron-transport stack, whereas the shorter TRPL lifetime indicates accelerated carrier recombination arising from additional nonradiative decay pathways. Consequently, the PL and TRPL results are complementary rather than contradictory and together provide a comprehensive picture of the carrier dynamics in the investigated devices. This interpretation is consistent with the EIS and photovoltaic results discussed in the following section, where the AZO-13-based devices exhibit a larger charge-transfer resistance together with lower JSC, FF, and PCE values. Similar relationships between PL quenching, carrier lifetime, and interfacial charge-transfer processes have been widely reported for perovskite solar cells employing different oxide electron-transport layers and interface engineering strategies.^84^

**Figure 9:**
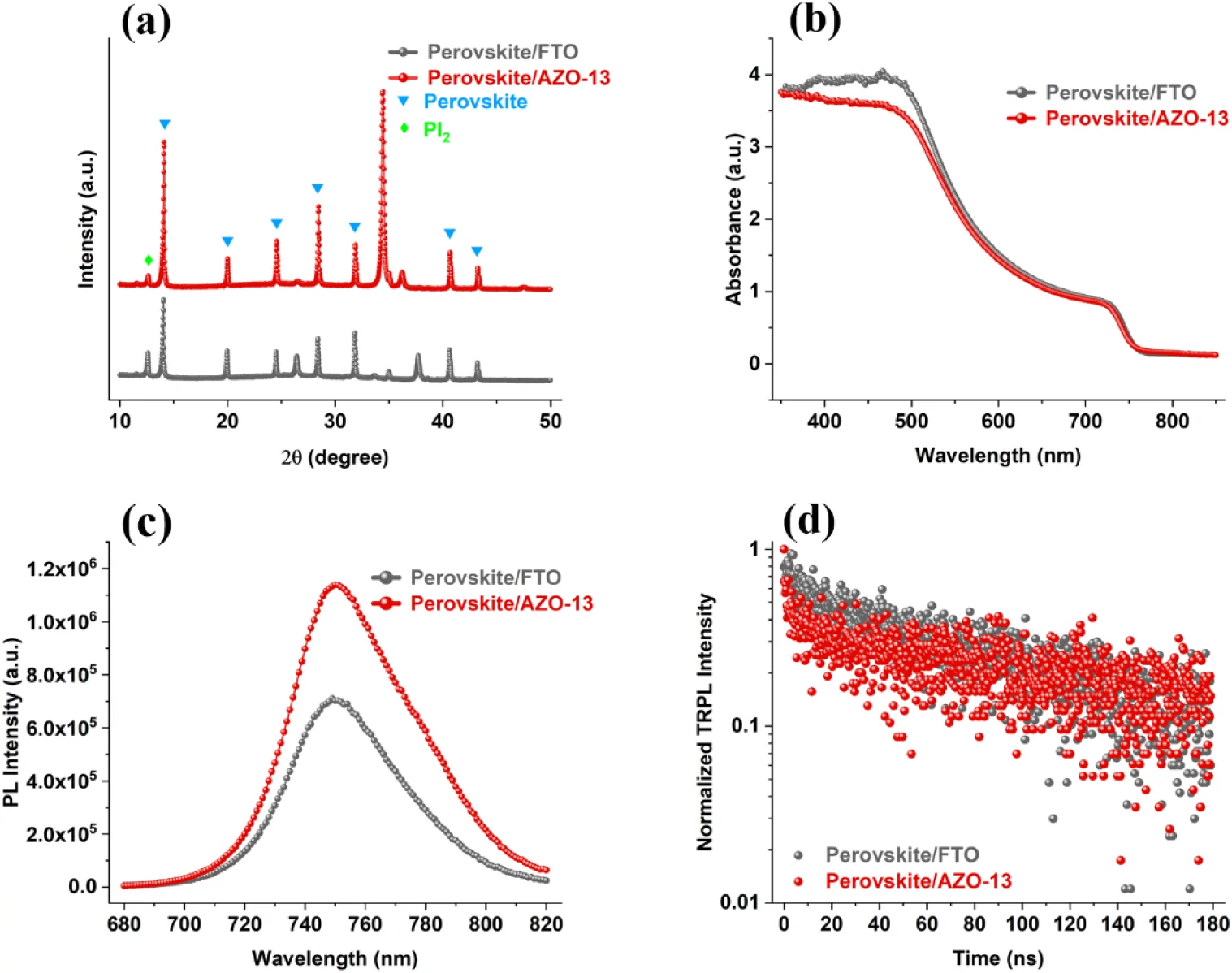
Structural and optoelectronic characterization of perovskite films deposited on compact TiO_2_-coated FTO and AZO-13 electrodes. (a) XRD patterns of perovskite/FTO and perovskite/AZO-13 structures, (b) optical absorbance spectra of the perovskite layers, (c) steady-state photoluminescence (PL) spectra of perovskite films grown on FTO and AZO-13 electrodes, and (d) TRPL decay curves of the perovskite layers.

**Table 2:** Time-Resolved Photoluminescence Fitting Parameters of Perovskite Films Deposited on Commercial FTO and Optimized AZO-13^a^

Sample	A_1_ (%)	τ_1_ (ns)	A_2_ (%)	τ_2_ (ns)	τ_av_ (ns)	χ^2^
Perovskite/FTO	0.30	2.09	99.70	200	199	1.03
Perovskite/AZO-13	2.63	2.70	97.37	131	128	0.98

a The table summarizes the biexponential fitting components, including the relative amplitudes (A_1_, A_2_), fast and slow decay lifetimes (τ_1_, τ_2_), average carrier lifetime (τ_av_), and fitting quality parameter (χ^2^), used to evaluate the interfacial charge-transfer dynamics and carrier recombination behavior of the perovskite layers.

This interpretation is further supported by subsequent device characterization. The significantly larger charge-transfer resistance obtained from the EIS measurements together with the lower JSC, FF, and PCE values of the PSC/AZO-13 devices consistently indicate inferior interfacial charge transport compared to the FTO-based devices. Collectively, the PL, TRPL, EIS, and photovoltaic results demonstrate that despite the improved structural quality of the AZO-13 electrode itself, the perovskite/c-TiO_2_/AZO-13 interface remains less effective for charge extraction than its FTO counterpart.

The surface morphology and topographical characteristics of the perovskite films are presented in Figure 10. The FESEM images shown in Figure 10a and Figure 10b reveal that both perovskite/FTO and perovskite/AZO-13 films possess similar polycrystalline morphologies with compact grain distributions and continuous surface coverage. In both structures, densely packed polygonal grains are observed, confirming successful perovskite crystallization on both TCO substrates. Several bright contrast regions are also visible, particularly in the lower-magnification images, and are likely associated with residual PbI_2_-rich secondary phases, consistent with the weak additional diffraction feature near ∼13° in Figure 9a. Similar PbI_2_-related segregations have frequently been reported in solution-processed perovskite films.^85,86^ The AFM images shown in Figure 10c and Figure 10d further confirm that both films exhibit comparable surface morphologies over the 2 μm × 2 μm scan area. Nevertheless, the perovskite/AZO-13 film exhibits slightly broader grain features and relatively more uniform surface topography compared with perovskite/FTO. In both cases, both substrates support the formation of continuous polycrystalline perovskite layers with compact surface coverage.

**Figure 10:**
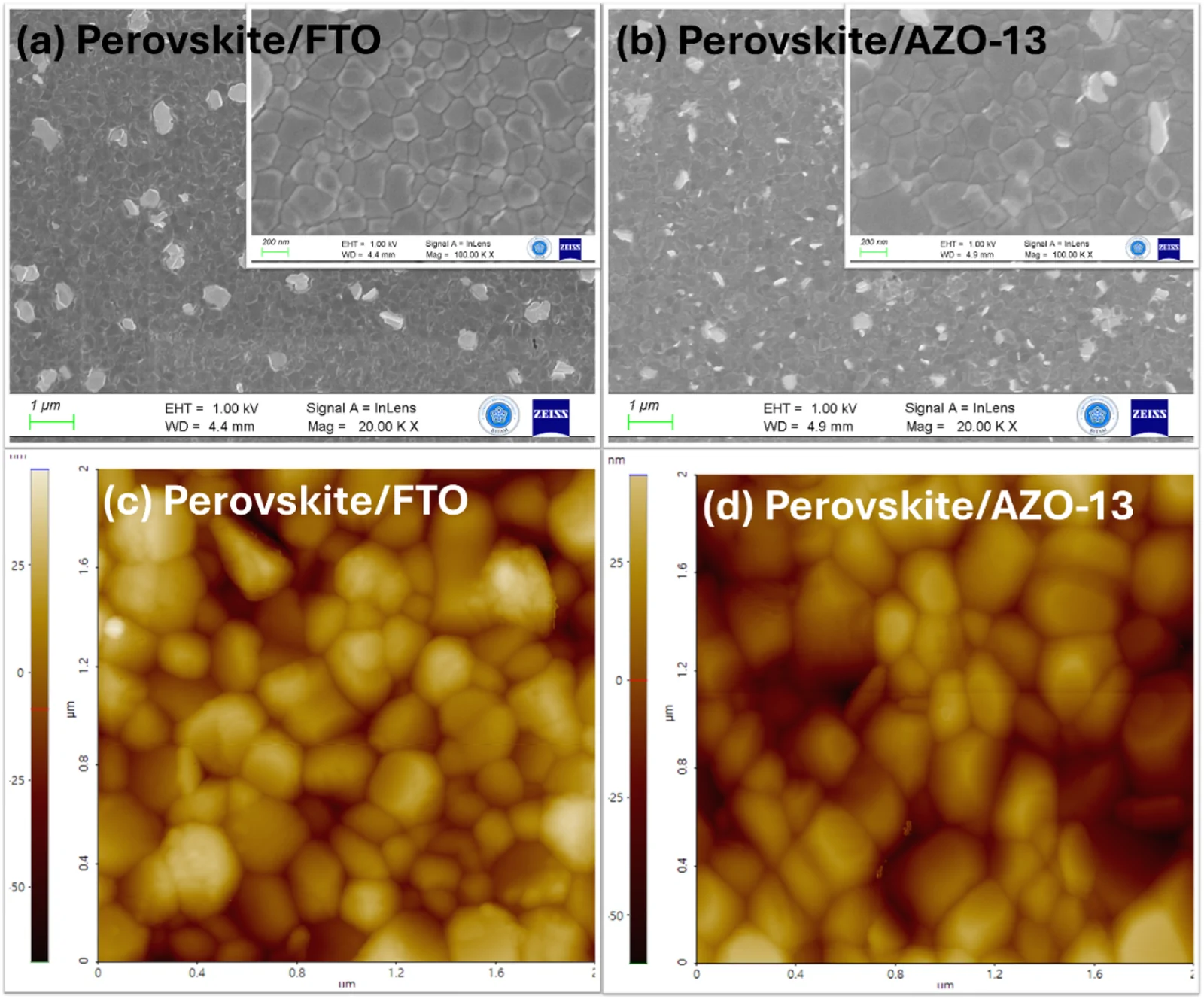
Comparative FESEM and AFM surface morphology analysis of perovskite on commercial FTO and optimized AZO-13 TCOs. (a) FESEM images of the perovskite/FTO and (b) perovskite/AZO-13 thin film recorded at magnifications of 20 kX and 100 kX. (c) AFM topography image of the perovskite/FTO substrate and (d) perovskite/AZO-13 thin film measured over a 2 μm × 2 μm scan area.

### Device Performance Analysis of FTO- and AZO-13-Based PSCs

The photovoltaic performance of PSC devices fabricated on commercial FTO and optimized AZO-13 electrodes was comparatively evaluated using J–V, EQE, EIS, and Maximum Power Point (MPP) analyses, as presented in Figure 11 and Table 3. The PSC/FTO device exhibited higher *V*_OC_, FF, and PCE values than PSC/AZO-13. Nevertheless, the AZO-13-based device exhibited stable photovoltaic operation despite using AZO instead of conventional FTO. The lower performance of PSC/AZO-13 is mainly attributed to its higher resistance and less efficient charge extraction at the interface. Similar relationships between TCO electrical properties and PSC performance have been widely reported for oxide-based transparent electrodes in perovskite solar cells.^87,88^ Although the stabilized efficiency of 16.49% obtained for the AZO-13-based PSC in this study remains below the higher efficiencies exceeding 18.5–19.4% recently reported for pulsed-laser-deposited AZO transparent electrodes in optimized PSC architectures,^27^ it falls within a practically relevant range for sputtered, fully indium-free AZO-based PSCs fabricated under standard planar processing conditions.

**Figure 11:**
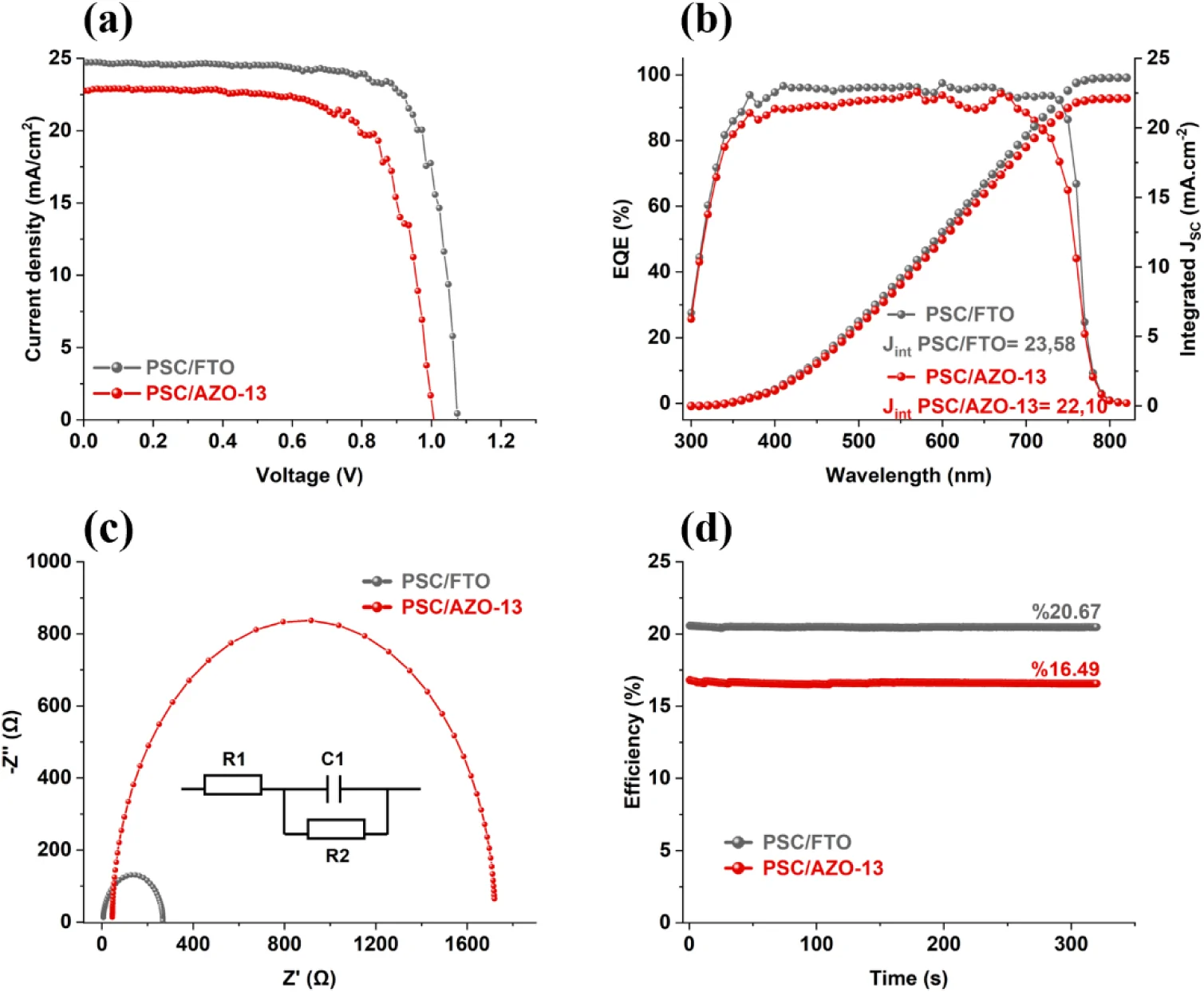
Comparative photovoltaic characterization of PSC devices fabricated on commercial FTO and optimized AZO-13 electrodes: (a) J–V curves, (b) EQE and integrated current density spectra, (c) EIS Nyquist plots, and (d) MPP tracking measurements.

**Table 3:** Photovoltaic Performance Parameters of PSC Devices Fabricated on Commercial FTO and Optimized AZO-13 Electrodes^a^

**Solar Cell**	***J***_**SC**_ **(mA·cm** ^**–2**^)		***V***_**OC**_ **(V)**	**FF** **(%)**	**PCE (%)**
**PSC/FTO**	**Champion**	24.7	1.08	78.4	20.7
	**Average**	24.6 ± 0.1	1.07 ± 0.02	77.2 ± 0.9	20.6 ± 0.1
**PSC/AZO-13**	**Champion**	22.8	1.01	72.0	16.5
	**Average**	22.9 ± 0.1	1.01 ± 0.01	69.4 ± 1.6	16.1 ± 0.3

a The reported values correspond to the average performance obtained from six individual pixels for PSC/FTO and five individual pixels for PSC/AZO-13 devices.

As shown in Figure 11a, both devices exhibited typical diode-like J–V behavior under illumination. PSC/FTO showed higher FF and sharper current decay near the open-circuit region, indicating more efficient charge transport and lower resistive losses, whereas PSC/AZO-13 exhibited increased transport resistance and interfacial recombination. Similar behavior has previously been associated with the conductivity and surface quality of the underlying TCO layer.^81,89^ The EQE and integrated current density spectra shown in Figure 11b further confirmed the photovoltaic response of both devices. PSC/FTO exhibited slightly higher EQE values and a larger integrated current density (J_int_ ≈ 23.58 mA·cm^–2^) compared with PSC/AZO-13 (J_int_ ≈ 22.10 mA·cm^–2^), suggesting more efficient carrier collection and reduced recombination losses. The Nyquist plots shown in Figure 11c revealed a substantially larger semicircle radius for PSC/AZO-13, suggesting higher charge-transfer resistance and increased recombination losses. PSC/FTO exhibited a charge-transfer resistance of 255 Ω and a series resistance of 7.24 Ω, whereas PSC/AZO-13 showed a much larger charge-transfer resistance of 1690 Ω. Similar EIS behavior has previously been associated with enhanced charge transport and reduced recombination in high-efficiency PSCs.^90^ The MPP tracking results shown in Figure 11d demonstrated stable operational behavior for both devices, with stabilized efficiencies of approximately 20.67% for PSC/FTO and 16.49% for PSC/AZO-13. Since all fabrication steps were identical, the performance difference can be directly attributed to the TCO layer properties. These results suggest that further improvements in AZO conductivity and interface quality may enable AZO-based PSCs to approach the performance of conventional FTO-based devices.

Figure 12 presents the statistical distribution of the photovoltaic parameters obtained from PSC devices fabricated on commercial FTO and optimized AZO-13 electrodes. As shown in Figure 12a–d, PSC/FTO devices exhibited consistently higher *J*_SC_, *V*_OC_, FF, and PCE than PSC/AZO-13 devices, indicating more efficient charge transport and reduced interfacial recombination. In contrast, PSC/AZO-13 devices exhibited lower average photovoltaic performance, which is consistent with the significantly higher charge-transfer resistance determined from the EIS analysis. To provide a quantitative interpretation of the impedance spectra, the experimental EIS data were fitted using the equivalent circuit model *R*_*s*_ + (*R*_*ct*_ ∥ *C*). The corresponding fitting parameters, together with the fitting quality, are summarized in Table S7 (Supporting Information), while the experimental and fitted Bode plots are presented in Figure S6 (Supporting Information). The equivalent-circuit fitting yielded a substantially higher *R_ct_* value for the PSC/AZO-13 device than for the PSC/FTO device, quantitatively confirming the less efficient interfacial charge-transfer characteristics of the AZO-based PSC. Nevertheless, the relatively narrow distribution of the photovoltaic parameters observed for PSC/AZO-13 demonstrates acceptable device reproducibility and confirms that sputtered AZO electrodes can serve as promising alternative TCO platforms for PSC applications after further optimization.

**Figure 12:**
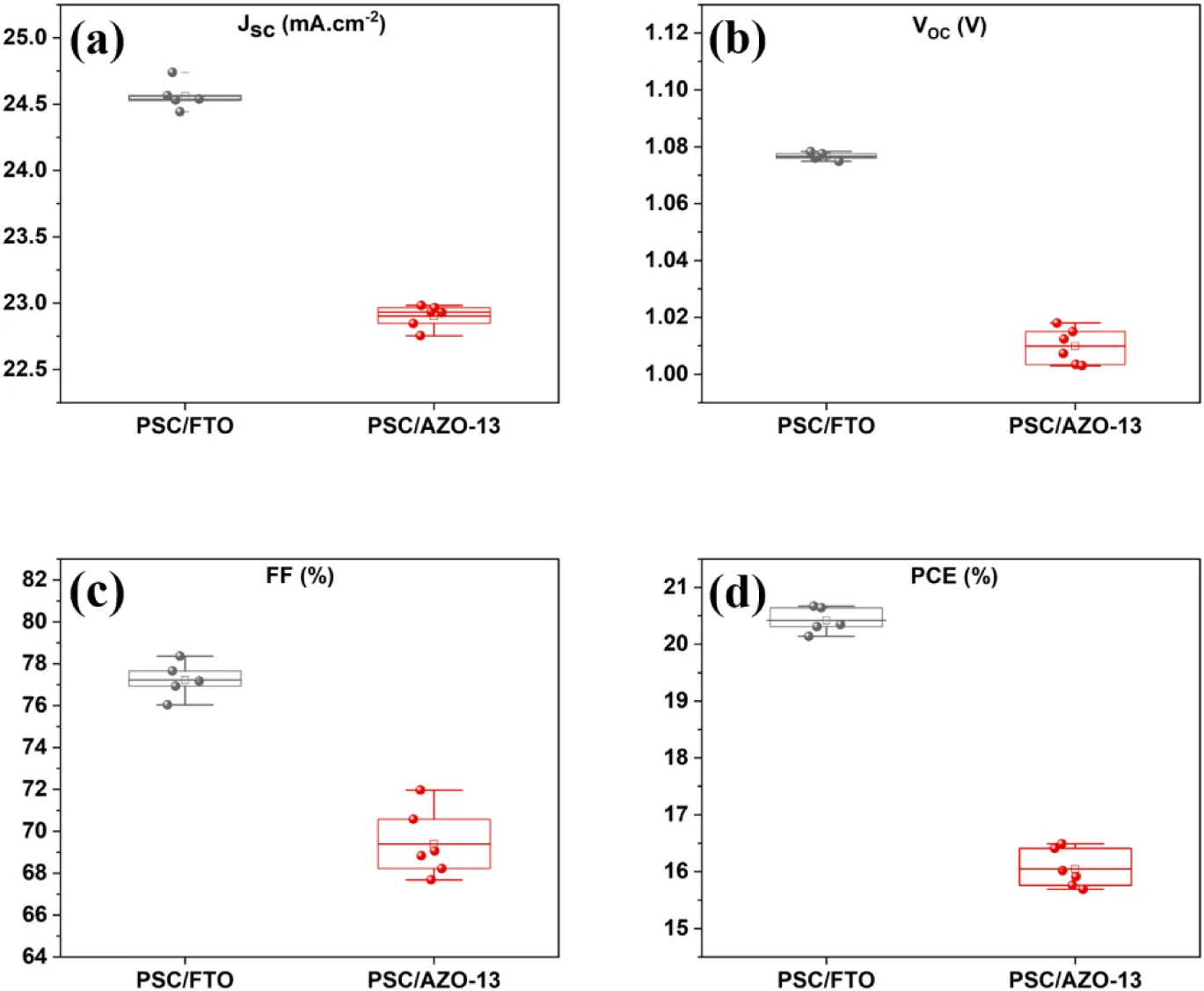
Statistical distribution of photovoltaic parameters for PSC devices fabricated on commercial FTO and optimized AZO-13 transparent conductive electrodes: (a) JSC, (b) VOC, (c) FF, and (d) PCE.

Taken together, the structural, optical, electrical, and photovoltaic characterization results consistently demonstrate that optimization of the sputtering conditions leads to simultaneous improvements in the properties of the AZO films. The enhanced crystallinity evidenced by the XRD results, together with the reduced structural disorder indicated by Raman spectroscopy, is expected to suppress grain-boundary and defect-related carrier scattering, thereby improving the electrical conductivity of the AZO electrode. The improved optoelectronic properties facilitate more efficient charge extraction at the electrode/perovskite interface, which is consistent with the PL/TRPL, EIS, and J–V results demonstrating enhanced carrier transport and photovoltaic performance for the optimized AZO-13 electrode.

## Conclusions

In summary, this study establishes a direct and quantitative link between sputtering process parameters, AZO thin-film properties, and PSC device performance through systematic optimization and side-by-side benchmarking against commercial FTO under identical fabrication conditions. Sequential control of sputtering power, postdeposition annealing, substrate temperature, and the power–time deposition balance progressively enhanced the film crystallinity, with the crystallite size increasing from 17.34 to 35.20 nm, and improved the electrical conductivity by more than 4 orders of magnitude, from 5.98 × 10^–2^ S·cm^–1^ for the as-deposited film to 9.85 × 10^2^ S·cm^–1^ for the optimized AZO-13 electrode. XPS analysis further confirmed that this improvement originates from enhanced substitutional incorporation of Al^3+^ into the ZnO lattice and an increased concentration of oxygen-deficiency-related donor states, without the formation of secondary phases. The optimized AZO-13 film combined an average visible transmittance of 86.0% with a Haacke figure of merit (0.668) slightly exceeding that of commercial FTO (0.664), demonstrating that sputtered, indium-free AZO electrodes can match or even exceed the transparency–conductivity balance of conventional TCOs.

When integrated into planar PSC devices fabricated under conditions identical to the FTO-based reference, AZO-13-based devices achieved a stabilized PCE of 16.49%, confirming that a systematically optimized AZO electrode can support stable and reasonably efficient perovskite photovoltaic operation. The performance gap relative to FTO-based devices (20.67%) was traced, through EIS and photoluminescence analysis, to a larger interfacial charge-transfer resistance at the perovskite/electrode interface rather than to any deficiency in the intrinsic optoelectronic quality of the AZO-13 film itself, pointing to a well-defined and addressable target for future interface engineering. Notably, AZO-13 exhibited enhanced transparency in the near-infrared region relative to that of FTO, a characteristic of particular relevance for semitransparent and tandem photovoltaic architectures, where efficient long-wavelength photon transmission to the underlying subcell is essential.

A limitation of the present study is that the AZO optimization and PSC benchmarking were restricted to a single planar device architecture and a single laboratory-scale fabrication line. Taken together, these results establish a clear process–structure–property–device relationship for sputtered AZO electrodes and provide a validated framework for their further development as low-cost, indium-free, and scalable alternatives to commercial TCOs in next-generation photovoltaic technologies. However, because the optimized AZO electrode was fabricated using a high-temperature deposition process, its direct integration into temperature-sensitive device architectures, such as flexible PSCs and completed monolithic tandem devices, would require the development of low-temperature deposition routes. Building on the interfacial charge-transfer limitation identified here, future work should focus on interface engineering strategies, alternative PSC architectures, and tandem photovoltaic configurations to fully translate the favorable material-level properties of optimized AZO electrodes into competitive device-level performance.

## Supplementary Material



## Notes

Hall effect parameters of selected AZO thin films and commercial FTO measured at room temperature under a magnetic field of 7210 G (Table S1); structural parameters of the AZO thin films calculated from the ZnO (002) diffraction peak, including crystallite size (*D*), lattice parameter (*c*), microstrain (ε), dislocation density (δ), residual strain/stress values, texture coefficient *TC­(002)*, and relative (002) orientation fraction (Table S2); thickness, average visible transmittance (*T*_*av*_) (400–800 nm), optical bandgap (*E*_*g*_), sheet resistance (R_S_), electrical resistivity (ρ), and electrical conductivity (σ) of the sputtered AZO thin films deposited under different sputtering conditions, together with the corresponding values of the commercial FTO substrate used as the reference transparent conducting electrode (Table S3); average visible transmittance (*T*_*av*_), sheet resistance (R_S_), and Haacke high-resolution figure of merit (FOM­(H-HR)) of the sputtered AZO thin films deposited under different sputtering conditions, together with the commercial fluorine-doped tin oxide (FTO) substrate used as the reference transparent conducting electrode (Table S4); fitted XPS peak parameters (binding energy, fwhm, relative peak area, and coefficient of determination, R^2^) for the Zn 2p, Al 2p, and O 1s (O_L_, O_D_) spectra of the AZO-1 and AZO-13 thin films (Table S5); surface roughness parameters of the optimized AZO-13 thin film and the commercial FTO substrate obtained from AFM measurements, including root-mean-square roughness (Rq), average roughness (Ra), maximum peak height (Rvp), maximum peak-to-valley height (Rz), and skewness (Rsk) (Table S6); equivalent circuit fitting parameters obtained from electrochemical impedance spectroscopy (EIS) analyses of PSC/FTO and PSC/AZO-13 devices using the equivalent circuit model *R*_*s*_ + (*R*_*ct*_ ∥ *C*) (Table S7);photograph of the Hall effect measurement setup showing the van der Pauw contact configuration used for the electrical characterization of the AZO thin films and the commercial FTO substrate (Figure S1); optical profilometry image and step-height analysis of the optimized AZO-13 thin film used for thickness determination in the Tauc plot analysis (Figure S2); current–voltage (I–V) characteristics and corresponding resistance values of AZO thin films (Figure S3); photograph of the Ag contact geometry with two Ag electrodes separated by 5 mm, used for the current–voltage measurements of AZO-1–AZO-14 thin films deposited under different sputtering conditions during the optimization process (Figure S4); XPS survey spectra of the AZO-1 (reference) and AZO-13 (optimized) thin films, confirming the presence of Zn, Al, and O together with the adventitious C 1s signal used for charge correction (Figure S5); experimental and fitted Bode plots of the electrochemical impedance spectroscopy (EIS) spectra for the (a) PSC/FTO and (b) PSC/AZO-13 devices (Figure S6) (PDF)
