# Supplementary material for: Systematic Optimization of Sputtered AZO Thin Films and Their Integration as Alternative Transparent Electrodes for Perovskite Solar Cells
Source: ACS Omega. 2026 Aug 21;11(34):51931–50. doi: 10.1021/acsomega.6c06694 (PMC13625117; doi:10.1021/acsomega.6c06694)
Supplement: Supplementary file 1 [file ao6c06694_si_001.pdf]

SUPPORTING INFORMATION

Systematic Optimization of Sputtered AZO Thin Films and Their Integration as Alternative Transparent Electrodes for Perovskite Solar Cells

Zeynep Mavili[†,‖], *Mucahit Yilmaz*[*,†,§]

[†] Laboratory of Advanced Materials & Photovoltaics (LAMPs), Necmettin Erbakan University, Konya, 42090, Türkiye.

[§] Department of Basic Sciences, Necmettin Erbakan University, Konya, 42090, Türkiye

[‖] Department of Nanoscience and Nanoengineering, Institute of Science, Necmettin Erbakan University, Konya 42090, Türkiye

[*] Corresponding Author: Mucahit Yilmaz

mucahityilmaz@erbakan.edu.tr

Table S1. Hall effect parameters of selected AZO thin films and commercial FTO measured at room temperature under a magnetic field of 7210 G.

**Sample Name**	**Conductivity Type**	**Carrier Concentration, *n***	**Mobility, *μ***	**Sheet Resistance, *Rs***	**Resistivity, *ρ***
		**(cm^{-3})**	**($cm^2.V.^{-1}.s^{-1}$)**	**($\Omega.sq^{-1}$)**	**(Ω.cm)**
AZO-1	n-type	1.13×10^{20}	0.99	1.85×10^{4}	5.56×10^{-2}
AZO-7	n-type	8.74×10^{20}	0.17	1.39×10^{4}	4.18×10^{-2}
AZO-11	n-type	2.45×10^{21}	1.70	5.99×10^{1}	1.50×10^{-3}
AZO-13	n-type	2.12×10^{21}	1.59	2.29×10^{1}	1.85×10^{-3}
AZO-14	n-type	6.52×10^{20}	5.60	2.85×10^{1}	1.71×10^{-3}
FTO	n-type	2.70×10^{20}	30.74	1.37×10^{1}	7.52×10^{-4}

Table S2. Structural parameters of the AZO thin films calculated from the ZnO (002) diffraction peak, including crystallite size (*D*), lattice parameter (*c*), microstrain (ε), dislocation density (δ), residual strain/stress values, texture coefficient *TC(002)*, and relative (002) orientation fraction.

Sample Name	*D* (nm)	*c* (Å)	ε ($\times 10^{-3}$)	δ ($\times 10^{15} m^{-2}$)	*c*-strain (%)	Stress (gPa)	*TC(002)*	$F_{(002)}$
AZO-1	17.34	5.1775	2.088	3.325	-0.559	1.300	2.726	0.850
AZO-2	17.76	5.1755	2.039	3.172	-0.597	1.390	2.838	0.906
AZO-3	14.85	5.1670	2.437	4.532	-0.761	1.771	2.817	0.885
AZO-4	17.73	5.1465	2.042	3.182	-1.154	2.686	2.889	0.931
AZO-5	15.59	5.1643	2.322	4.114	-0.813	1.892	2.801	0.885
AZO-6	20.17	5.1496	1.795	2.459	-1.095	2.550	2.893	0.935
AZO-7	17.58	5.1833	2.059	3.235	-0.448	1.043	2.883	0.927
AZO-8	13.27	5.1593	2.728	5.680	-0.909	2.115	2.903	0.933
AZO-9	16.09	5.2237	2.250	3.863	0.329	-0.766	2.811	0.874
AZO-10	24.92	5.1840	1.453	1.611	-0.434	1.011	2.991	0.994
AZO-11	30.78	5.1950	1.176	1.055	-0.223	0.520	2.970	0.978
AZO-12	24.69	5.1557	1.466	1.640	-0.977	2.275	2.968	0.981
AZO-13	35.20	5.1623	1.029	0.807	-0.850	1.979	2.995	0.997
AZO-14	32.06	5.1613	1.129	0.973	-0.870	2.026	2.994	0.996

Table S3. Thickness, average visible transmittance (T_{av}) (400–800 nm), optical bandgap (E_g), sheet resistance (Rs), electrical resistivity (ρ), and electrical conductivity (σ) of the sputtered AZO thin films deposited under different sputtering conditions, together with the corresponding values of the commercial FTO substrate used as the reference transparent conducting electrode. The sheet resistance values of the AZO films were obtained from the measured current–voltage (I–V) characteristics using two parallel Ag electrodes separated by 5 mm with an electrode width of 5 mm (L=W), while the resistivity and conductivity were calculated according to $\rho = Rs.t$ where t is thickness and $\sigma = 1/\rho$, respectively.

Sample Name	Thickness (nm)	T_{av}(400-800 nm) (%)	E_g (eV)	Rs ($\Omega.sq^{-1}$)	ρ (Ω.cm)	σ ($S.cm^{-1}$)
AZO-1	107	77.4	3.07	1.56×10^{6}	1.67×10^{1}	5.98×10^{-2}
AZO-2	104	63.7	3.18	7.97×10^{5}	8.29×10^{0}	1.21×10^{-1}
AZO-3	102	73.6	3.24	3.52×10^{3}	3.59×10^{-2}	2.79×10^{1}
AZO-4	92	62.6	3.21	4.04×10^{3}	3.72×10^{-2}	2.69×10^{1}
AZO-5	67	73.5	3.31	2.82×10^{3}	1.89×10^{-2}	5.30×10^{1}
AZO-6	76	76.7	3.33	3.69×10^{2}	2.81×10^{-3}	3.56×10^{2}
AZO-7	79	78.3	3.35	3.05×10^{2}	2.41×10^{-3}	4.16×10^{2}
AZO-8	168	78.8	3.28	5.07×10^{2}	8.52×10^{-3}	1.17×10^{2}
AZO-9	189	80.6	3.20	5.11×10^{2}	9.65×10^{-3}	1.04×10^{2}
AZO-10	205	81.4	3.43	6.36×10^{1}	1.30×10^{-3}	7.67×10^{2}
AZO-11	207	78.3	3.36	3.53×10^{1}	7.30×10^{-4}	1.37×10^{3}
AZO-12	206	83.6	3.48	5.49×10^{1}	1.13×10^{-3}	8.85×10^{2}
AZO-13	807	86.0	3.45	1.26×10^{1}	1.01×10^{-3}	9.85×10^{2}
AZO-14	758	74.8	3.33	1.68×10^{1}	1.28×10^{-3}	7.84×10^{2}
FTO	550	82.0	3.90	8.23×10^{0}	4.53×10^{-4}	2.21×10^{3}

Table S4. Average visible transmittance (T_{av}), sheet resistance (Rs), and Haacke high-resolution figure of merit (FOM(H-HR)) of the sputtered AZO thin films deposited under different sputtering conditions, together with the commercial fluorine-doped tin oxide (FTO) substrate used as the reference transparent conducting electrode. The FOM(H-HR) values were calculated according to the Haacke high-resolution model[70-72], $\Phi_{H-HR} = T_{av} / Rs^{1/10}$.

**Sample Name**	**T_{av}(400-800 nm) (%)**	**Rs ($\Omega.sq^{-1}$)**	**FOM(H-HR)**
AZO-1	77.4	1.56×10^{6}	0.186
AZO-2	63.7	7.97×10^{5}	0.164
AZO-3	73.6	3.52×10^{3}	0.325
AZO-4	62.6	4.04×10^{3}	0.273
AZO-5	73.5	2.82×10^{3}	0.332
AZO-6	76.7	3.69×10^{2}	0.25
AZO-7	78.3	3.05×10^{2}	0.442
AZO-8	78.8	5.07×10^{2}	0.423
AZO-9	80.6	5.11×10^{2}	0.432
AZO-10	81.4	6.36×10^{1}	0.537
AZO-11	78.3	3.53×10^{1}	0.548
AZO-12	83.6	5.49×10^{1}	0.560
AZO-13	86.0	1.26×10^{1}	0.668
AZO-14	74.8	1.68×10^{1}	0.564
FTO	82.0	8.23×10^{0}	0.664

Table S5. Fitted XPS peak parameters (binding energy, FWHM, relative peak area, and coefficient of determination, R^2) for the Zn2p, Al2p, and O1s (O_L, O_D) spectra of the AZO-1 and AZO-13 thin films.

Sample Name	Peak	Center (eV)	FWHM (eV)	Area (%)	R^2
AZO-1	$Zn2p_{3/2}$	1021.27	1.94	67.10	0.998
AZO-13	$Zn2p_{3/2}$	1021.39	2.12	66.50	0.992
AZO-1	$Zn2p_{1/2}$	1044.37	1.98	32.90	0.998
AZO-13	$Zn2p_{1/2}$	1044.59	2.18	33.50	0.992
AZO-1	Al2p	73.86	1.69	100	0.996
AZO-13	Al2p	74.22	1.77	100	0.994
AZO-1	O1s - O_L	529.96	1.41	35.14	0.995
AZO-13	O1s - O_L	530.05	1.41	31.90	0.999
AZO-1	O1s - O_D	531.04	2.56	64.86	0.995
AZO-13	O1s - O_D	531.50	2.45	68.10	0.999

Table S6. Surface roughness parameters of the optimized AZO-13 thin film and the commercial FTO substrate obtained from AFM measurements, including root-mean-square roughness (Rq), average roughness (Ra), maximum peak height (Rvp), maximum peak-to-valley height (Rz), and skewness (Rsk).

Sample Name	Rq (nm)	Ra (nm)	Rvp (nm)	Rz (nm)	Rsk
AZO-13	8.753	7.087	55.565	54.506	0.037
FTO	9.375	7.537	53.553	52.470	-0.210

Table S7. Equivalent circuit fitting parameters obtained from electrochemical impedance spectroscopy (EIS) analyses of PSC/FTO and PSC/AZO-13 devices using the equivalent circuit model $R_s + (R_{ct} \parallel C)$. The corresponding chi-square (χ^2) values indicate the quality of the fitting.

**Parameter**	**PSC/FTO Values**	**PSC/AZO-13 Values**	**Unit**
R1 (Rs)	7.24×10^{0}	4.59×10^{1}	Ω
R2 (Rct)	2.55×10^{2}	1.69×10^{3}	Ω
C1	2.20×10^{-8}	2.98×10^{-8}	F
χ^2	2.16×10^{-3}	6.26×10^{-2}	

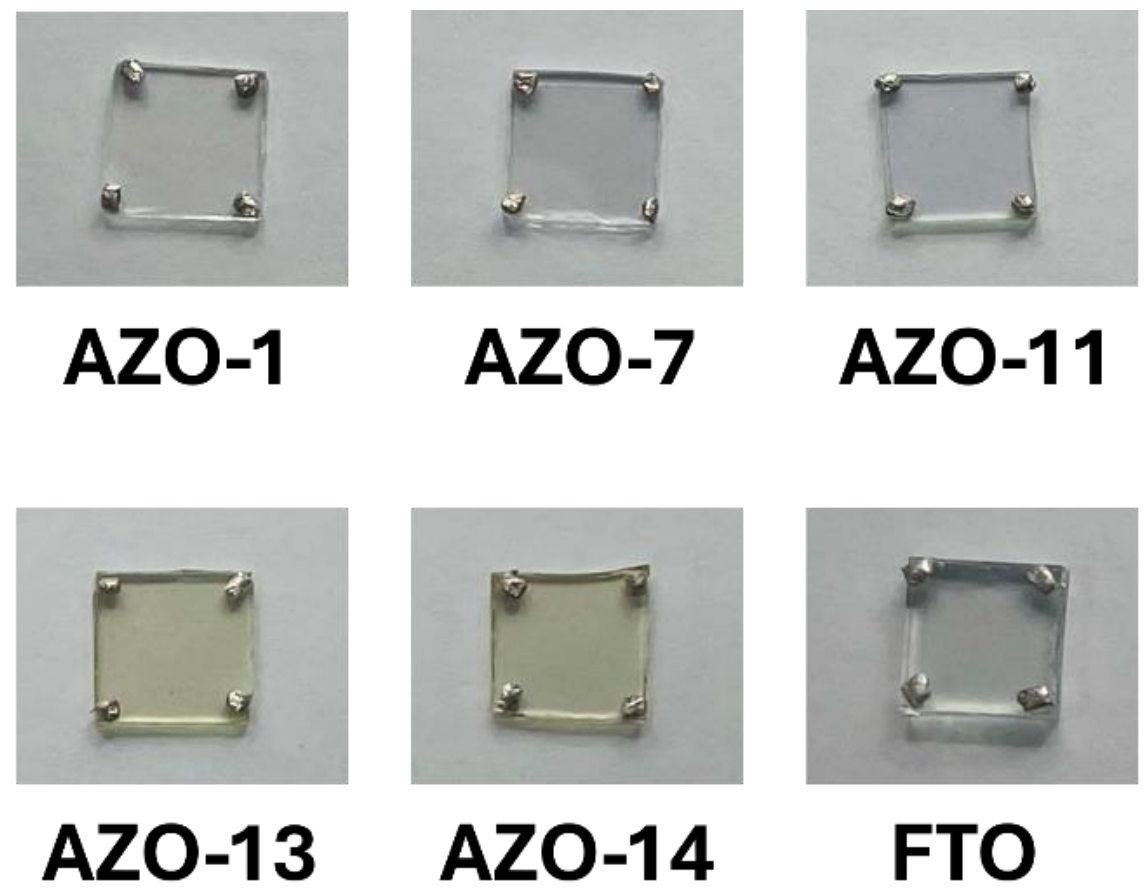

Figure S1. Photograph of the Hall effect measurement setup showing the van der Pauw contact configuration used for the electrical characterization of the AZO thin films and the commercial FTO substrate.

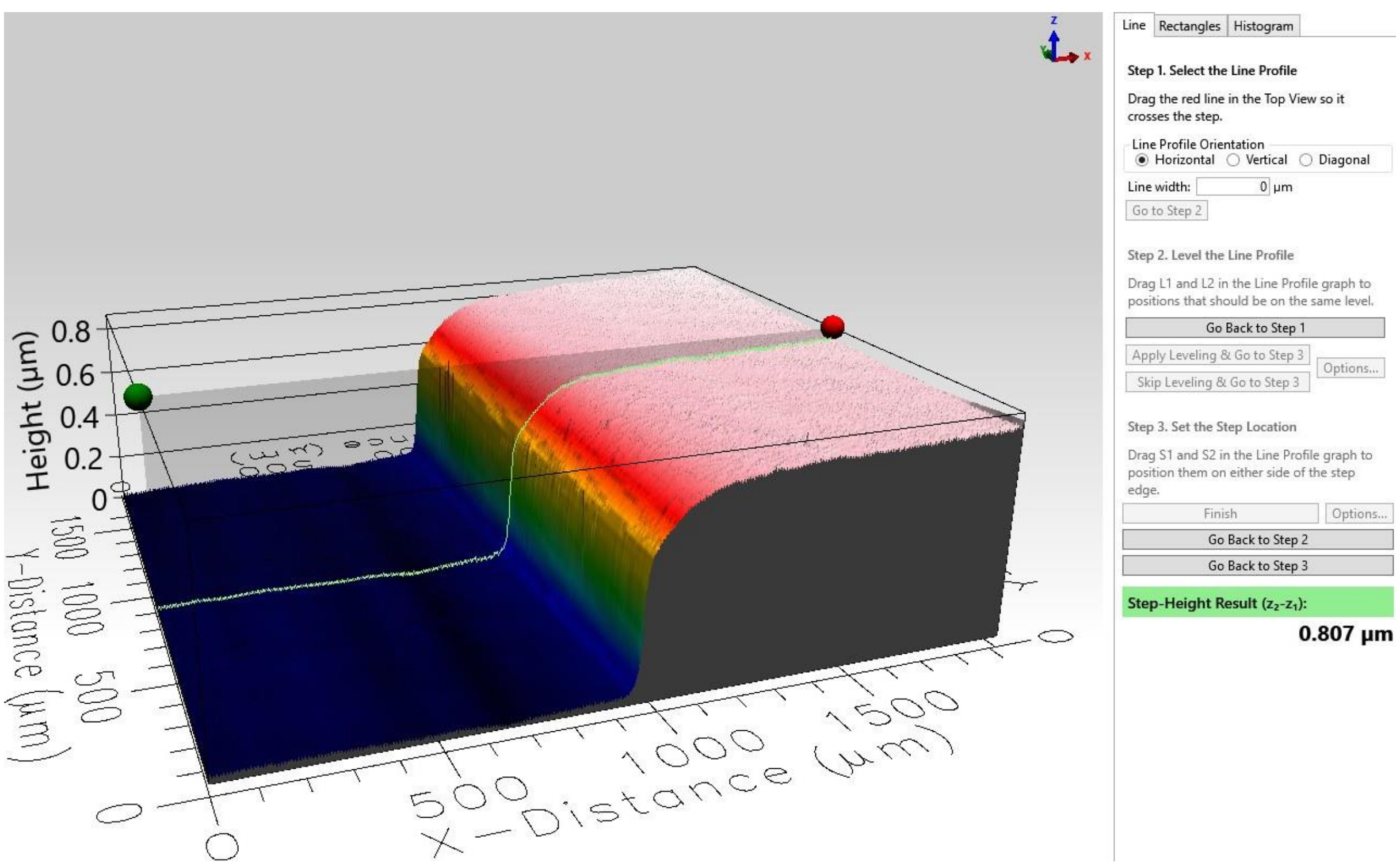

Figure S2. Optical profilometry image and step-height analysis of the optimized AZO-13 thin film used for thickness determination in the Tauc plot analysis. The measured film thickness was approximately 0.807 μm.

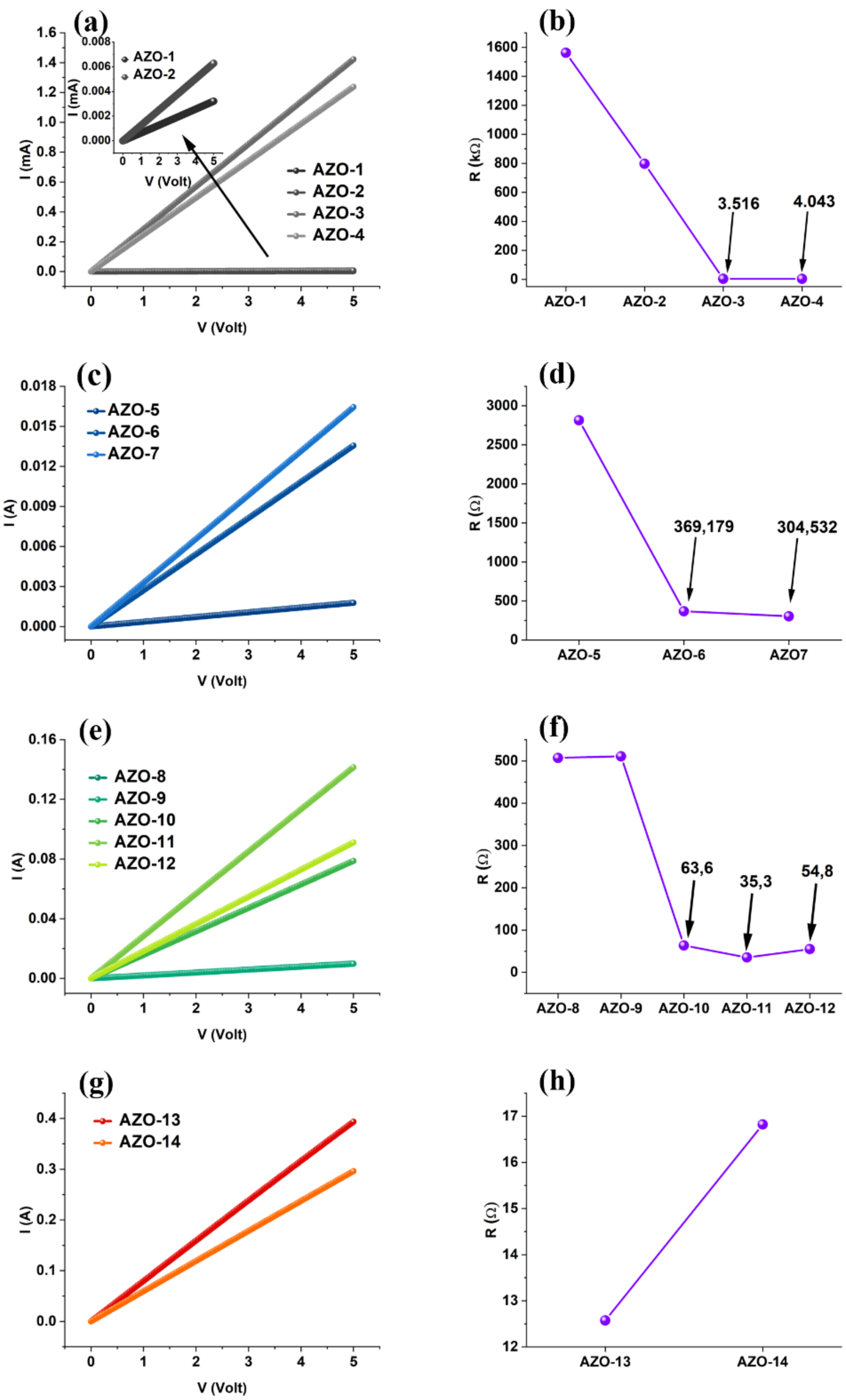

Figure S3. Current–voltage (I–V) characteristics and corresponding resistance values of AZO thin films. (a), (b) room-temperature-grown power series (AZO-1–AZO-4); (c), (d) post-annealed films (AZO-5–AZO-7); (e), (f) substrate-heated films (AZO-8–AZO-12); (g), (h) final optimized films AZO-13 and AZO-14.

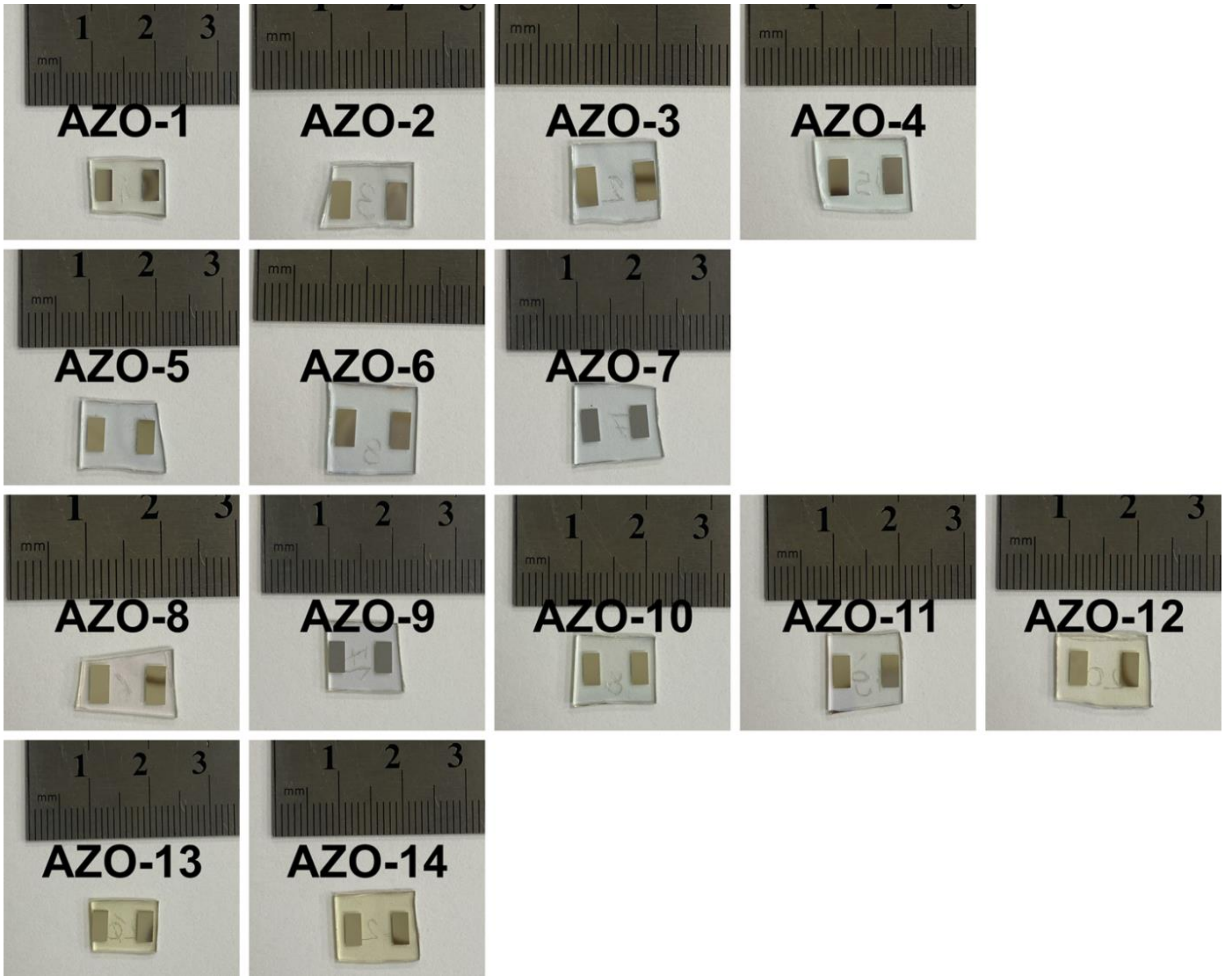

Figure S4. Photograph of the Ag contact geometry with two Ag electrodes separated by 5 mm, used for the current–voltage measurements of AZO-1–AZO-14 thin films deposited under different sputtering conditions during the optimization process.

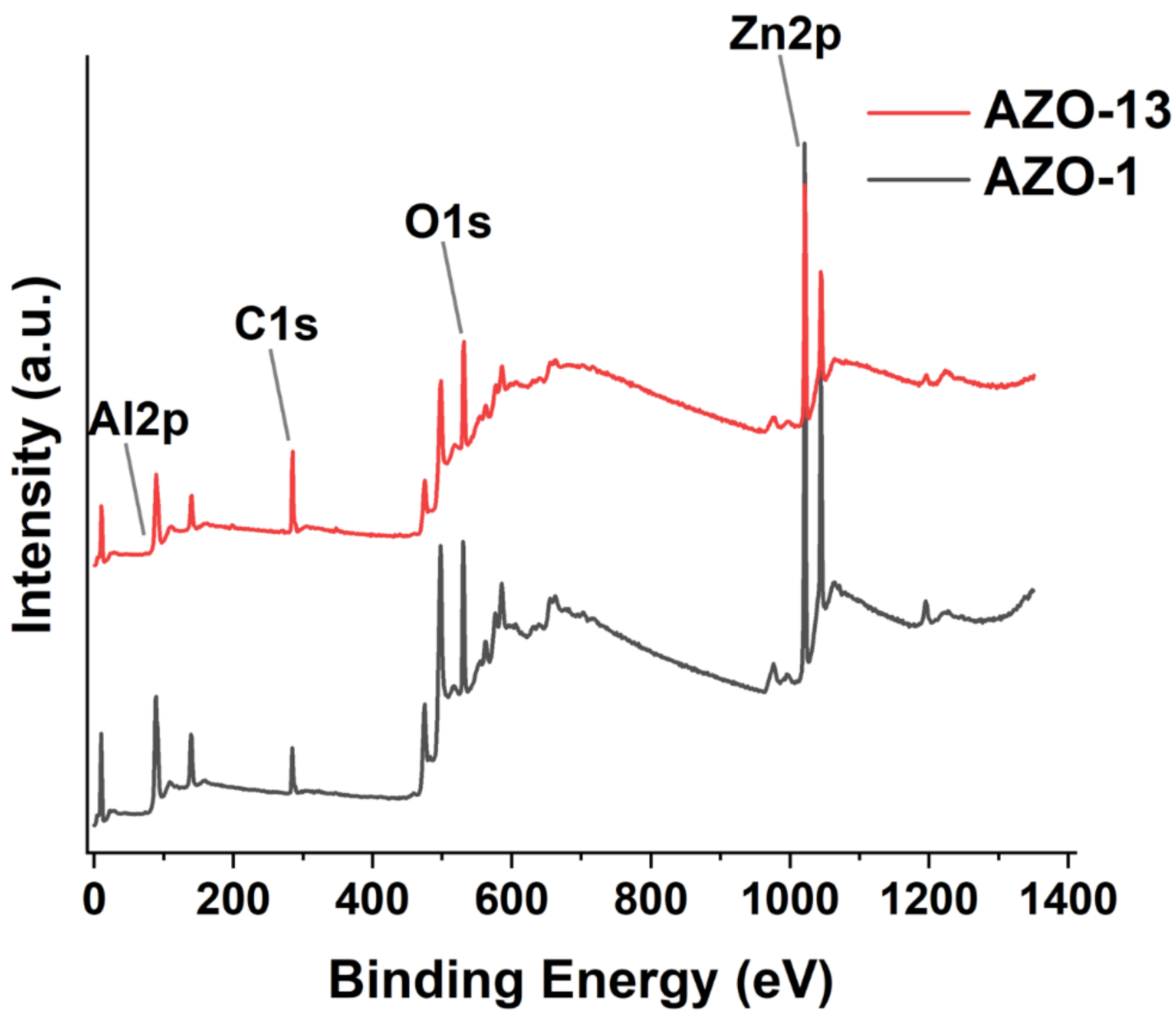

Figure S5. XPS survey spectra of the AZO-1 (reference) and AZO-13 (optimized) thin films, confirming the presence of Zn, Al, and O together with the adventitious C1s signal used for charge correction.

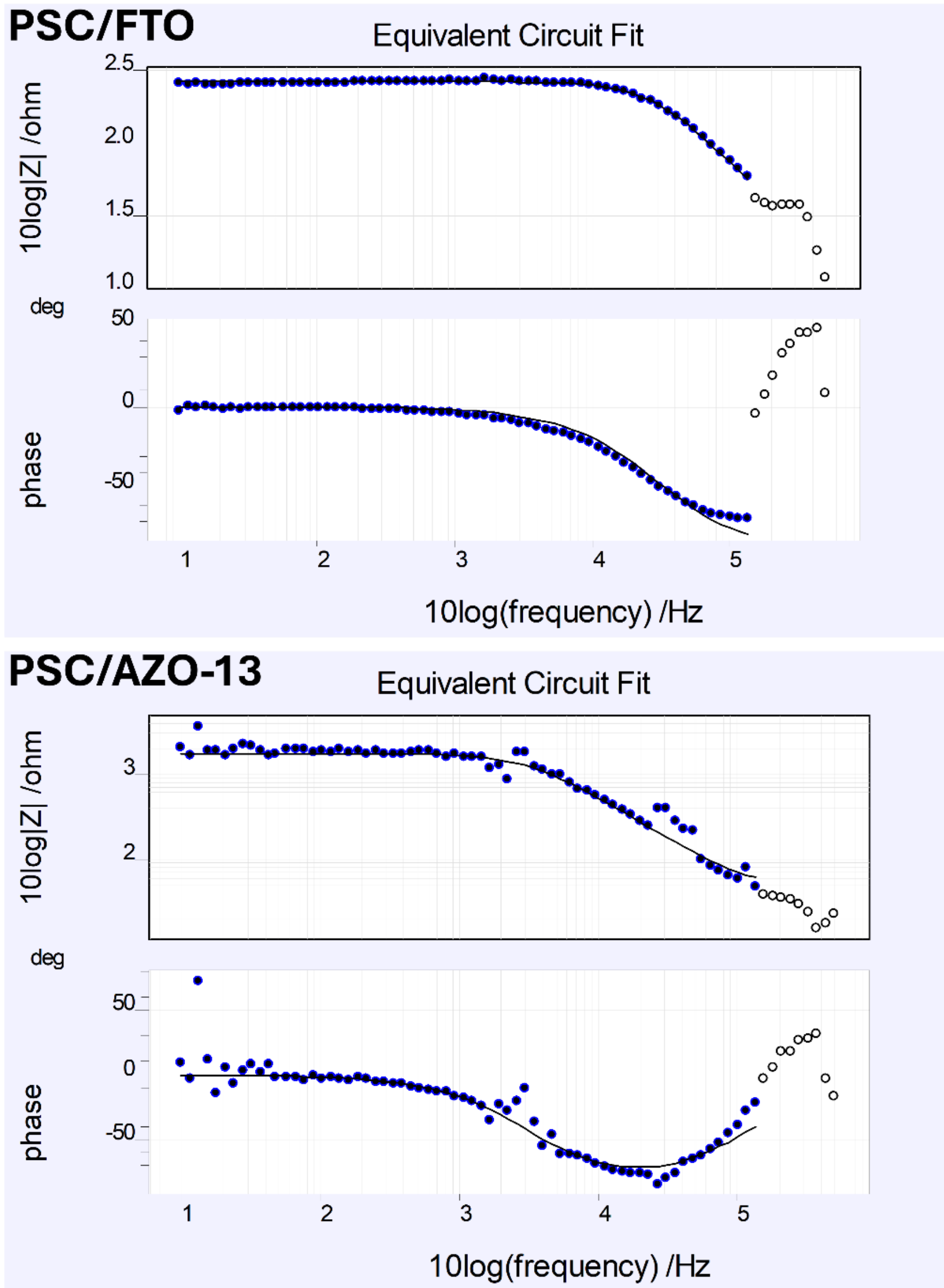

Figure S6. Experimental and fitted Bode plots of the electrochemical impedance spectroscopy (EIS) spectra for the (a) PSC/FTO and (b) PSC/AZO-13 devices. The impedance data were analyzed using the equivalent circuit model $R_s + (R_{ct} \parallel C)$, where $R_s$denotes the series resistance, $R_{ct}$the charge-transfer resistance, and C the interfacial capacitance. Symbols indicate the experimental data, and solid lines represent the fitted results.
